# CDK-driven phosphorylation of TRAIP is essential for mitotic replisome disassembly and MiDAS

**DOI:** 10.1093/nar/gkaf530

**Published:** 2025-07-10

**Authors:** Divyasree Poovathumkadavil, Alicja Reynolds-Winczura, Aggeliki Skagia, Paolo Passaretti, Martina Muste Sadurni, Georgia Kingsley, Alexander Leitner, Marco Saponaro, Agnieszka Gambus

**Affiliations:** Department of Cancer and Genomic Sciences, Birmingham Centre for Genome Biology, University of Birmingham, Birmingham, B15 2TT, United Kingdom; Department of Cancer and Genomic Sciences, Birmingham Centre for Genome Biology, University of Birmingham, Birmingham, B15 2TT, United Kingdom; Department of Cancer and Genomic Sciences, Birmingham Centre for Genome Biology, University of Birmingham, Birmingham, B15 2TT, United Kingdom; Department of Cancer and Genomic Sciences, Birmingham Centre for Genome Biology, University of Birmingham, Birmingham, B15 2TT, United Kingdom; Department of Cancer and Genomic Sciences, Birmingham Centre for Genome Biology, University of Birmingham, Birmingham, B15 2TT, United Kingdom; Department of Cancer and Genomic Sciences, Birmingham Centre for Genome Biology, University of Birmingham, Birmingham, B15 2TT, United Kingdom; Department of Biology, Institute of Molecular Systems Biology, ETH Zurich, Zurich, 8093, Switzerland; Department of Cancer and Genomic Sciences, Birmingham Centre for Genome Biology, University of Birmingham, Birmingham, B15 2TT, United Kingdom; Department of Cancer and Genomic Sciences, Birmingham Centre for Genome Biology, University of Birmingham, Birmingham, B15 2TT, United Kingdom

## Abstract

Disassembly of the replication machinery (replisome) from chromatin is an active process driven by two ubiquitin ligases Cul2^LRR1^ and TRAIP, which both target the Mcm7 subunit of the replicative helicase for ubiquitylation. Uncontrolled unloading of replisomes during S-phase would be disastrous for genome stability and cell viability. On the other hand, replisomes retained on under-replicated DNA in mitosis require removal to allow access and processing of the DNA before cell division. TRAIP ubiquitylates replisomes in mitosis but can also act in specific situations during S-phase. However, we do not know how TRAIP’s activity is regulated to stop uncontrolled replisome unloading. Here we show that TRAIP activity towards replisomes is not regulated at the level of interaction with the substrate: it interacts with terminated replisomes in S-phase without ubiquitylation. However, in mitosis, TRAIP is phosphorylated by cyclin-dependent kinases (CDKs) and this phosphorylation is essential for mitotic replisome unloading. CDK phosphorylation of TRAIP stimulates its autoubiquitylation activity and ubiquitylation of replisomes isolated from mitotic chromatin. The phosphorylation of TRAIP is also important in human cells for TRAIP functions during MiDAS. Although essential during mitosis, the CDK-driven phosphorylation of TRAIP is not sufficient to activate uncontrolled unloading of replisomes in S-phase.

## Graphical Abstract

**Graphical Abstract:**
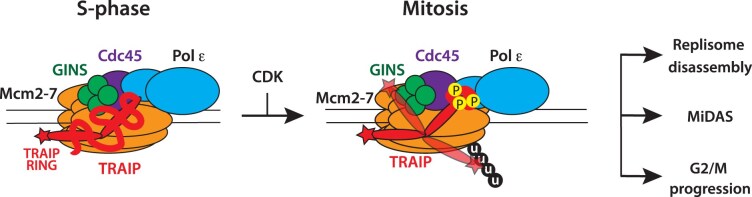

## Introduction

DNA replication occurs in three stages: initiation, elongation, and termination. In human cells, DNA replication initiates from thousands of replication origins. These are the positions within the genome where replicative helicases become activated and start unwinding DNA while moving in opposite directions, away from each other, creating two DNA replication forks. The replicative helicase is composed of Cdc45, Mcm2–7 hexamer, and GINS complex (CMG complex) [1]; it is positioned at the tip of replication forks and forms a platform for replisome assembly [2]. Once established, the replication forks replicate chromatin until they encounter forks coming in opposite directions from neighbouring origins. At this point, the termination of replication forks takes place. The removal of the replisome from fully duplicated DNA is the last stage of fork termination [3] and is driven by Cul2^LRR1^ ubiquitin ligase, which ubiquitylates Mcm7 within the terminated CMG helicase complex [4, 5]. Such modified CMG is then recognized by p97 (VCP) segregase and removed from chromatin allowing for disassembly of the whole replisome built around the helicase [5–7]. Crucially, any helicase complexes that fail to be unloaded in S-phase are alternatively unloaded in mitosis [4, 8]. The disassembly of the post-termination replisome retained on chromatin during mitosis is independent of cullin-type ubiquitin ligases but requires TRAIP ubiquitin ligase activity and p97 segregase function [9, 10].

DNA replication forks often become stressed in a variety of circumstances during S-phase and fail to terminate [11]. Moreover, not all DNA is always replicated during S-phase: unreplicated DNA is detected in many cancer cells in mitosis; DNA synthesis can proceed during mitosis (mitotic DNA synthesis, MiDAS); while under-replicated DNA can lead to the formation of ultrafine bridges in anaphase and formation of structures in G1 stage of the next cell cycle that are bound by 53BP1 protein (53BP1 bodies) [12–14]. Genome-wide, such unreplicated regions correlate with common fragile sites (CFS), which are chromosomal loci responsible for the majority of rearrangements found in human disease [15]. When replication forks cannot complete replication during S-phase, these forks and their associated replisomes are retained on chromatin until mitosis and become the substrate for TRAIP ubiquitylation leading to replisome disassembly [9, 10]. The mitotic pathway is therefore not just a backup pathway for the disassembly of terminated replisomes, but is essential to remove any replisome from chromatin before cell division, and TRAIP activity is required for cells to be able to deliver MiDAS [16].

Apart from these important mitotic functions, TRAIP activity is also essential during S-phase, and indeed we have shown that rapid degradation of auxin-induced degron of TRAIP (TRAIP-mAID) at different stages of the cell cycle specifically inhibited proliferation of cells when TRAIP was degraded in S-phase [17]. Without TRAIP, cells progressing through S-phase could not resolve replication–transcription collisions at promoters with bidirectional transcription [17]. TRAIP was also reported over the years to be required for appropriate repair of DNA damage caused by mitomycin C (MMC), camptothecin (CPT), UV, and hydroxyurea (HU), as well as for translesion DNA synthesis [18–21], and was proposed as a master regulator of interstrand cross-link repair through differential ubiquitylation of the replisome [22].

Uncontrolled replisome unloading during S-phase would be disastrous for genome stability and cell viability as the mechanisms to ensure one-per-cell-cycle DNA replication inhibit re-loading of the helicase onto already established replication forks. The S-phase checkpoint mechanisms are known to safeguard stability of the replisome at the stalled forks during S-phase allowing for its unloading only when it is necessary for processing of DNA by repair mechanisms [11]. It is essential, therefore, that CMG unloading is strictly controlled and prohibited at active replication forks. Cul2^LRR1^ ubiquitin ligase activity towards terminated replisomes is regulated at the level of ligase interaction with the substrate. The main LRR1 interaction surface within the replisome is obscured by the lagging strand protruding from the active replicative helicase, and only when this obstruction is gone, e.g. when the terminated replisomes stop unwinding DNA and slide onto a double-stranded DNA of last Okazaki fragment, the Cul2^LRR1^ can bind and ubiquitylate Mcm7 within the terminated replisome [23–25]. However, the regulation of TRAIP activity towards the replisomes in S-phase and in mitosis is not known. In human cells, TRAIP is expressed ubiquitously throughout the entire cell cycle, peaking during G2/M [26]. TRAIP has been shown previously to reside predominantly in the nucleolus due to sumoylation [27] and re-localize to sites of DNA damage due to C-terminal PIP box [18, 19], but was also shown to interact with nascent DNA in unperturbed S-phase through nascent chromatin capture (NCC) [18, 26] and the PIP box of TRAIP is not essential for its activity [18]. In *Xenopus* egg extract, TRAIP was also proposed to be a part of unchallenged replication fork [9]. Altogether, unlike Cul2^LRR1^, the regulation of TRAIP activity towards the replisome does not seem to be just simply regulated at the level of ubiquitin ligase–substrate interaction. However, it still has to be tightly regulated to avoid premature replisome unloading and resulting genome instability. This requirement is supported by observations whereby even moderate levels of TRAIP overexpression were reported to be cytotoxic, while epitope tagging of TRAIP (especially at the N-terminus) affects its functionality in the cells [18]. Moreover, mutations of TRAIP ubiquitin ligase activity in humans lead to microcephalic primordial dwarfism [19], with some of the mutations reported affecting other than ubiquitin ligase catalytic activity.

We have set off, therefore, to analyse TRAIP’s chromatin binding, interaction with the replisome, Mcm7 ubiquitylation, and replisome unloading across S-phase and mitosis, so that we can determine the stimuli that regulate TRAIP activity towards the replisome during different stages of the cell cycle. By doing so, we found that TRAIP interacts with terminated or stalled replisomes during S-phase, but its activity towards such replisomes is subdued. TRAIP does not contribute to the unloading of terminated replisomes in S-phase. However, in mitosis, TRAIP is phosphorylated by cyclin-dependent kinases (CDKs), and this phosphorylation is essential for mitotic replisome unloading. CDK phosphorylation of TRAIP stimulated autoubiquitylation activity of TRAIP *in vitro*, and ubiquitylation of replisomes isolated from mitotic chromatin. The phosphorylation of TRAIP is also important in human cells for TRAIP functions during MiDAS. Although essential during mitosis, the CDK-driven phosphorylation of TRAIP is not sufficient to activate uncontrolled unloading of replisomes in S-phase.

## Materials and methods

### *Xenopus laevis* egg extract preparation

All of the work with *Xenopus laevis* was approved by Animal Welfare and Ethical Review Body at University of Birmingham and approved by UK Home Office in form of Project License issued for Prof. Agnieszka Gambus. *Xenopus laevis* egg extract was prepared as previously described [28].

### Recombinant proteins

Recombinant His/SUMO-tagged *X. laevis* wild type (wt), 3A, 3D, and 3E mutants of TRAIP were expressed in Rosetta (DE3) pLysS cells from pCA528 vector O/N at 18°C in AIM media supplemented with 50 μM ZnSO_4_. Frozen bacterial pellets were lysed in resuspension buffer (20 mM HEPES–NaOH, pH 7.5, 400 mM NaCl, 10% glycerol, 10 μM ZnSO_4_, 0.1% NP-40, 1 mM dithiothreitol (DTT), 0.1 mM phenylmenthanesulfonyl fluoride (PMSF), and 1× Roche cOmplete protease inhibitor cocktail), supplemented with 1 mg/ml lysozyme and 25 U/ml BaseMuncher and incubated at 4°C for 20 min. After sonication, the lysate was clarified by centrifugation at 14 000 × *g* for 30 min at 4°C, and supernatants containing soluble proteins were then incubated with 2 ml of pre-washed Super Ni-NTA Affinity Resin (SUPER-NINTA100, Generon) for 1 h, rotating at 4°C. After a centrifugation at 1000 rpm for 3 min, the depleted fraction was collected and incubated again with fresh 2 ml of pre-washed Super Ni-NTA Affinity Resin for 1 h, rotating at 4°C. The beads from both rounds were then washed 3× with Wash Buffer (20 mM HEPES–NaOH, pH 7.5, 400 mM NaCl, 10% glycerol, 20 mM imidazole, 10 μM ZnSO_4_, 0.01% NP-40, 1 mM DTT, and 1× Roche cOmplete protease inhibitor cocktail). Resin-bound wt and mutants of TRAIP were eluted using Elution Buffer (20 mM HEPES–NaOH, pH 7.5, 400 mM NaCl, 10% glycerol, 250 mM imidazole, 0.01% NP-40, and 1 mM DTT). Those fractions containing the highest quantities of wt or mutants of TRAIP were dialysed into LFB1/100 buffer (2 mM DTT, 1 mM Ethylene glycol-bis(β-aminoethylether)-N,N,N′,N′-tetraacetic acid (EGTA), 40 mM HEPES–KOH, pH 8, 100 mM KCl, 20 mM KH_2_PO_4_, pH 8, 2 mM MgCl_2_, 10% sucrose). The dialysed fractions were added dropwise into a pool of liquid nitrogen and the frozen beads stored at −80°C.

Recombinant His-tagged ubiquitin and UbiNOK were purchased from Boston Biochem (U-530-02M and UM-HNOK), dissolved in LFB1/50 buffer (10% sucrose, 50 mM KCl, 40 mM HEPES, pH 8.0, 20 mM K phosphate, pH 8.0, 2 mM MgCl_2_, 1 mM EGTA, 2 mM DTT, 1 μg/ml of each aprotinin, leupeptin, and pepstatin), and used at 0.5 mg/ml in the extract.

### Inhibitors

MLN4924 (A01139, Active Biochem) was dissolved in 5 mM Dimethylsulfoxide (DMSO) and added to the extract 15 min after addition of sperm chromatin at 10 μM. NMS873 (17674, Cayman Chemical Company) was dissolved in DMSO at 10 mM and added to the extract 15 min after addition of sperm nuclei at 50 μM. Caffeine (C8960, Sigma) was dissolved in water at 100 mM and added to the extract along with demembranated sperm chromatin at 5 mM. Aphidicolin was dissolved in DMSO at 8 mM and added to the extract along with demembranated sperm chromatin at 40 μM. UbiVS (U-212-025, Biotechne) was added to the extract at a final concentration of 10 μM along with demembranated sperm chromatin.

### Antibodies

Anti-PCNA (P8825) was purchased from Sigma–Aldrich, anti-Vinculin 7F9 (sc-73614) was purchased from Santa Cruz, α-Cul2 (EPR3104) was purchased from Abcam, and α-TRAIP (NBP1-87125) was purchased from Novus Biologicals. Affinity-purified anti-Cdc45, anti-Psf2 [29], anti-Mcm7 [10], anti-GINS antibody [30], and anti-TRAIP [17] were previously described.

### TRAIP immunodepletion

TRAIP immunodepletions were performed using Dynabeads Protein A (10002D, Life Technologies) coupled to *Xenopus* TRAIP antibodies raised in rabbit and affinity-purified or non-specific rabbit IgG (I5006, Sigma). The immunodepletion of TRAIP using purified antibodies required coupling of 600 μg antibodies to 1 ml of Dynabeads following manufacturer's protocol. Effective immunodepletion required two rounds of 1-h incubation of egg extract with antibody-coupled beads at 50% bead ratio (e.g. two rounds of 100 μl of egg extract incubated with 50 μl of coupled beads).

### DNA synthesis assay

Interphase *X. laevis* egg extract was supplemented with 10 ng/μl of demembranated sperm nuclei, indicated treatment as described below and α^32^P-dATP (PerkinElmer). The extract was incubated at 23°C for indicated time. Synthesis of nascent DNA was then measured by quantification of α^32^P-dATP incorporation into newly synthesized DNA, as described before [28]. In summary, the reaction was stopped, and DNA was trichloroacetic acid precipitated and finally filtered through glass fibre filters. Filters were washed in ethanol and dried, and precipitated radioactivity then counted in scintillation liquid.

The extract contains endogenous deoxynucleoside triphosphate pools of 50 μM [31]. The total amount of DNA synthesized expressed as ng DNA/μl extract can then be calculated by multiplying percent total ^32^P incorporated by a factor of 0.654 [31]. This calculation assumes an average molecular weight of 327 Da for deoxynucleoside monophosphate and equal quantities of all four deoxynucleoside triphosphates incorporated into DNA (weight of deoxynucleoside monophosphate incorporated in ng/μl = percent total ^32^P incorporated/ 100 × 50 × 10^−6^ × 4 × 327 × 10^3^) [31].

### Chromatin isolation time course

Interphase *X. laevis* egg extract was supplemented with 10 ng*/*μl of demembranated sperm DNA and appropriate recombinant proteins or inhibitors. Recombinant TRAIP purified in this study was added at 6 μM. The reaction was incubated at 23°C for indicated time and chromatin was isolated in ANIB100 buffer (50 mM HEPES, pH 7.6, 100 mM KOAc, 10 mM MgOAc, 2.5 mM Mg-ATP, 0.5 mM spermidine, 0.3 mM spermine, 1 μg/ml of each aprotinin, leupeptin and pepstatin, 25 mM β-glycerophosphate) supplemented with 0.1% Triton X-100, 0.1 mM PMSF, and 10 mM 2-chloroacetamide as previously described [28]. A control sample without addition of sperm DNA (no DNA) was processed in an analogous way during the procedure, to serve as a chromatin specificity control. Chromatin samples were run in SDS–PAGE gel and the bottom of the gel was cut off and incubated with Colloidal Coomassie (SimplyBlue, Life Technologies) to stain histones, serving as loading controls and indications of sample contamination with egg extract (cytoplasm). Chromatin-bound proteins were then analysed by western blotting using indicated antibodies.

### Immunoprecipitation from chromatin

*Xenopus laevis* egg extract was activated into interphase by adding energy regenerator (50 mM HEPES, pH 7.6, 306 mg/ml creatine phosphate, 3.6 mg/ml creatine phosphokinase), 0.25 mg/ml cycloheximide, and 0.3 mM CaCl_2_. Extract was split into four aliquots and supplemented with 40 μM aphidicolin and 5 mM caffeine, 50 μM NMS873, 10 μM MLN4924, and control with LFB 1/50 (40 mM HEPES–KOH, pH 8, 20 mM KH_2_PO_4_, pH 8, 2 mM MgCl_2_, 50 mM KCl, 1 mM EGTA, 2 mM DTT, and 10% sucrose), respectively. Finally, each condition was supplemented with 10 ng/μl of demembranated sperm DNA and incubated at 23°C for 45-60 min. After incubation, chromatin was isolated in ANIB100-NO ATP (50 mM HEPES, pH 7.6, 100 mM KOAc, 10 mM MgOAc, 0.5 mM spermidine, 0.3 mM spermine, 1 μg/ml of each aprotinin, leupeptin and pepstatin, 25 mM β-glycerophosphate, and 0.1 mM Na_3_VO_4_) supplemented with 0.1% Triton X-100, 0.1 mM PMSF, and 10 mM 2-chloroacetamide, as previously described [28]. Isolated chromatin was then resuspended in the same volume of original extract of ANIB100-NO ATP containing 20% sucrose. Protein complexes were released from chromatin by digestion with 2 U/μl of Benzonase nuclease (E1014-25KU, Sigma) for 30 min at room temperature (RT) and sonication for 5 min using a Diagenode sonicator with settings: 15 s on/off, low setting. Sample was then spun in a microfuge at maximum speed for 5 min at 4°C to separate soluble and insoluble fractions.

Inputs of 380 μl each containing soluble chromatin protein complexes were incubated with 50 μl of Dynabeads M-270 epoxy (14301, Life Technologies) beads covalently coupled with GINS antibodies or non-specific IgG from sheep serum (I5131, Sigma). Input and beads were incubated at 4°C for 2 h with rotation. Following the incubation time, beads were washed twice with ANIB100-NO ATP, once with ANIB100-NO ATP containing 0.1% Triton X-100, and finally twice with ANIB100-NO ATP buffer, for 5 min rotating at 4°C each wash. Finally, IP proteins were eluted by boiling the beads in 50 μl of 2× NuPAGE LDS loading buffer (Life Technologies) for 5 min.

### *In vitro* ubiquitylation assay

Ubiquitylation assay was set up on ice in 20 μl reactions containing 50 nM E1 UBA1 (E-305-025, Boston Biochem), 0.5 μM E2 UbcH5a/UBE2D1 (E2-616-100, Boston Biochem), 50 μM His6-Ubiquitin (U-530-02M, Boston Biochem), 1 μM TRAIP, in the ubiquitylation reaction buffer (50 mM HEPES, pH 8.0, 150 mM NaCl, 5 mM MgCl_2_, 0.1% Triton X-100, 2 mM DTT, and 250 mM Mg-ATP). Control reactions with no E1, E2, or TRAIP were also set up in parallel. The mixtures were incubated at 37°C for 90 min shaking. The reactions were then stopped by adding 4× NuPAGE LDS loading buffer and boiling at 95°C for 5 min. The samples were finally run in a SDS–PAGE gel and analysed by western blotting using anti-TRAIP and anti-ubiquitin antibodies. At least two independently purified batches of each TRAIP-wt and mutants were tested in *in vitro* assays for reproducibility of results.

### *In vitro* kinase assay

*In vitro* kinase assay was performed based on [32]. Phosphorylation of recombinant TRAIP and its mutant versions was tested with CDK1/Cyclin B (PV3292; Thermo Scientific) and CDK2/Cyclin A (PV3267; Thermo Scientific). To test phosphorylation, an *in vitro* assay was set up with phosphor kinase buffer containing ATP, mitotic/S-phase recombinant CDKs at 0.35 μM final concentration, and recombinant purified TRAIP at a final concentration of 8 μM. The samples were incubated at 30°C for 30 min, and the reaction was analysed using a 6% SuperSep Phos-tag gel (192-17401; Fujifilm).

### Western blot quantification

Quantification of western blots is provided to show reproducibility of trends in experiments. The quantified experiments were performed in independently immunodepleted extracts or different preparations of extract to confirm that observed phenotypes are not dependent on an extract prep. Pixel densities for bands in the western blot and the scanned Coomassie-stained histones were quantified using ImageJ software. The values from each western blot band were normalized to the corresponding loading controls (bands of Coomassie-stained histones from the same sample). Analyses of control and treated samples were performed in parallel, and the fold difference between these samples was calculated. Data from replicate experiments were presented in a graph showing the fold change, along with the mean value and the standard error of the mean (SEM).

### Anion exchange, gel filtration, and glycerol gradient

For size-exclusion chromatography analyses, purified (as above) His6-SUMO-tagged *X. laevis* TRAIP-wt was first subjected to buffer exchange and further purified using a fast protein liquid chromatography (FPLC) system (AKTA go, Cytiva). Buffer exchange was performed using a 5-ml HiTrap desalting column (Cytiva). Anion exchange was performed using Mono Q anion exchange column (Cytiva). Protein was injected into the column in binding buffer (Tris–HCl 50 mM, pH 7.5, NaCl 2 M, EDTA 1 mM, DTT 1 mM, and PMSF 1 mM) to allow biding, and eluted with a linear gradient 0%–100% of elution buffer (Tris–HCl 50 mM, pH 7.5, EDTA 1 mM, DTT 1 mM, and PMSF 1 mM) over 12 ml. The eluted sample was collected on a 96-well plate in fractions of 250 μl. Fraction C1 with TRAIP-wt (Supplementary Fig. S5A) was then used for gel filtration and *in vitro* phosphorylation assay.

For size-exclusion chromatography analyses, TRAIP-wt and phosphorylated TRAIP-wt were centrifuged at 16 000 × *g* for 5 min. The supernatants were loaded one at a time into a FPLC system (AKTA go, Cytiva) using a 50 μl loop and injected into a Superdex 200 Increase 3.2/300 column (Cytiva). Gel filtration was carried on at 20 μl/min and sample collected in 50 μl fractions. For the SDS–PAGE, 10 μl of each fraction was loaded onto a NuPAGE™ Bis-Tris Mini Protein Gels, 4%–12% gradient gel (Invitrogen). After transferring to nitrocellulose membrane, western blot was performed using TRAIP antibodies.

Four millilitres of 20%–40% glycerol gradients in LFB1/100 (with no sucrose) buffer were prepared in 4-ml thin-wall tubes (Beckman Coulter, 328874). One Hundred microlitres of released chromatin protein sample was applied on top of the gradient and samples spun in an SW60 Ti rotor (Beckman Coulter) for 16 h at 45 000 rpm at 4°C. A separate glycerol gradient with size marker proteins was included in every run. After the spin, gradients were divided into 200 μl fractions and analysed by SDS–PAGE and western blotting as above for the gel filtration samples.

### Quantification of molecular size of TRAIP

The molecular sizes of analysed complexes were calculated according to Siegel and Monty [33] using the following size marker values: ferritin (440 kDa, 61 Å), aldolase (158 kDa, 48.1 Å, 7.4 S), conalbumin (75 kDa, 32 Å, 5.1 S), and ovalbumin (44 kDa, 3.6 S).

SUMO-TRAIP sedimentation coefficient was estimated at 5.105 S, while SUMO-TRAIP Stoke’s radius at 62.77 Å. Following Siegel and Monty’s formula


\begin{eqnarray*}
M = 6{\mathrm{\pi}} \eta NaS/\left( 1 - {vp} \right)
\end{eqnarray*}

[where *η* isviscosity of water (=1.02), *N* is Avogadro’s number (=6 × 10^23^); *a* is Stoke’s radius, *S* is the Sedimentation coefficient, *v* is partial specific volume (0.725 av), and *p* = density of water (1)], the calculated molecular size of TRAIP is 138 kDa.

### AlphaFold

The three-dimensional structure of TRAIP was predicted by using AlphaFold3 [34]. The amino acid sequence used as input was retrieved from the UniProt database (Q6NRV0). AlphaFold3 was accessed via AlphaFold server (https://alphafoldserver.com), and predictions were run with default parameters unless specified otherwise. The confidence of the predicted model was assessed using the per-residue predicted local distance difference test (pLDDT) scores, which were included in the output. Molecular graphics were performed with UCSF ChimeraX [35, 36].

### Sample preparation for XL-MS

In this study, TRAIP was analysed via crosslinking mass spectrometry (XL-MS) in two separate experiments. In the first one (Supplementary Fig. S3B), 50 μl of His6-SUMO-TRAIP-wt 7.5 μM in LFB 1/100 was cross-linked with 0.1 or 0.33 mM DSS-H_12_/D_12_ (Creative Molecules Inc.) for 30 min at RT. The reaction was quenched with 100 mM ammonium bicarbonate. Samples were dried in a vacuum centrifuge and redissolved in 8 M urea for reduction [2.5 mM tris(2-carboxyethyl)phosphine, 30 min, 37°C] and alkylation (5 mM iodoacetamide, 30 min, RT). Samples were diluted to ∼5.5 M urea with 150 mM ammonium bicarbonate and pre-digested with 200 ng endoproteinase Lys-C (Fujifilm Wako) for 2 h at 37°C, followed by further dilution to 1 M urea with 50 mM ammonium bicarbonate and digestion with 400 ng trypsin (Promega) at 37°C overnight. The following day, samples were acidified with formic acid to 2% (v/v) final concentration, and the digests were purified by solid-phase extraction using Sep-Pak tC18 cartridges (50 mg, Waters).

Aliquots of ∼500 ng of digests were analysed in duplicate by liquid chromatography–tandem mass spectrometry on a Thermo Orbitrap Fusion Lumos instrument connected to a Thermo Easy nLC-1200 system. Peptides were separated on a Thermo Acclaim PepMap C_18_ column (250 mm × 75 μm, 2 μm particle size) using a mobile-phase system of A = water/acetonitrile/formic acid (98:2:0.15, v/v/v) and B = acetonitrile/water/formic acid (80:20:0.15, v/v/v). The gradient was set from 11% B to 40% B over 60 min and the flow rate was 300 nl/min.

The mass spectrometer was operated in data-dependent acquisition mode with precursor selection according to the top speed mode with 3 s cycle time. Precursor ion spectra were acquired in the Orbitrap analyser at a resolution of 120 000, and candidates for sequencing were selected if they had a charge state between 3+ and 7+ and were not on a dynamic exclusion list (30 s after one sequencing event). Fragmentation was performed in the linear ion trap at 35% normalized collision energy, and fragment ion spectra were acquired in the Orbitrap analyzer at a resolution of 30 000.

Cross-linked peptide pairs were identified by xQuest, version 2.1.5 (available from gitlab.ethz.ch/leitner_lab/xquest_xprophet) [37, 38]. The database contained the sequence of the target protein, including the His-SUMO tag, and the most abundant contaminant proteins from the *Escherichia coli* expression system identified in a preliminary search. The following search parameters were used: enzyme = trypsin, maximum number of missed cleavages = 2, initial precursor mass error = 15 ppm, and fragment mass error = 15 ppm. Lys residues and protein N-termini were allowed as cross-linking sites, methionine oxidation was specified as a variable modification, while carbamidomethylation of cysteine was set as a fixed modification.

The primary search results were filtered according to the experimentally observed mass deviation (−5 to +1 ppm) and manually evaluated. No decoy hits were observed with a score cut-off of 25 after these filtering steps, so that the false discovery rate at the peptide-pair level is expected to be close to 0%. For presentation of the TRAIP cross-links in Supplementary Fig. S3B, cross-links from the tag region were removed and amino acid positions were adjusted to the wild-type sequence.

Similarly, in the second XL-MS experiment, TRAIP and phosphorylated TRAIP cross-linked peptides were compared (Supplementary Fig S3G and H). Phosphorylated TRAIP was prepared using 50 μl of His6-SUMO-TRAIP-wt 7.5 μM in LFB 1/100 to which CDK1/Cyclin B of 0.35 μM and ATP were added. The reaction was carried on for 30 min at 30°C. TRAIP and phosphorylated TRAIP were then cross-linked in LFB1/100 with 1 mM DSS-H_12_/D_12_ (Creative Molecules Inc.) for 30 min at RT, and the reaction was quenched with 100 mM ammonium bicarbonate.

Samples were acidified with formic acid to 2% (v/v) final concentration, and the digests were purified by solid-phase extraction using Sep-Pak tC18 cartridges (50 mg, Waters). Ten percent of the samples were used for direct mass spectrometric analysis, while the remainders were fractionated by size-exclusion chromatography using a Superdex 30 Increase column (3.2 mm × 300 mm, Cytiva) using a mobile phase of water/acetonitrile/trifluoroacetic acid (70:30:0.1, v/v/v) and a flow rate of 50 μl/min. Four fractions were collected and evaporated to dryness. Aliquots of ∼500 ng of digests (unfractionated of fractionated) were analysed in duplicate by liquid chromatography–tandem mass spectrometry on a Thermo Orbitrap Fusion Lumos instrument connected to a Thermo Easy nLC-1200 system.

Cross-links were plotted using xiVIEW [39], and cross-linked peptides were mapped onto the AlphaFold generated structures using ChimeraX and XMAS [40].

### Analysis of previously published mass spectrometry data

We re-analysed proteins identified in Mcm3 IP in S-phase (replisome retained on chromatin due to CULi and p97i treatment; published previously in [4]) and Mcm3 IP in mitosis (replisome retained on chromatin due to CULi and p97i treatment and mitosis induced through CylinA1ΔN addition; published previously in [10]). First, we compared total number of Mcm2–7 complex peptides identified in both experiments, to have the same base number of Mcm2–7 IPied in both experiments. We normalized number of peptides of all interactors identified in both experiments to the total Mcm2–7. Then, we calculated a fold difference in peptide numbers identified in mitosis/S-phase—the result is presented in Supplementary Table S1. Only proteins with at least 50 peptides identified in either dataset were considered.

We also searched through the previously published mass spectrometry data for identified phosphorylated peptides of TRAIP (Fig. 2). Number of TRAIP trypsin digestion peptides containing phosphorylation sites (as identified by Mascot and reported by Scaffold) as in either phosphorylated or non-phosphorylated form was counted. Only peptides with >90% confidence of phosphorylation identification were considered.

## Human cell line methods

### Cell lines and cell culture

HCT116 CMV-OsTIR1 TRAIP-mAID cell lines were modified through viral transduction to introduce plasmids containing either TRAIP-wt or TRAIP-3A mutant. Virus production and cell transduction was carried out as previously described in [17], for the generation of TRAIP rescue cell lines. All cell lines were grown in a humified incubator at 37°C and 5% CO_2_, in McCoy’s 5A modified media (Gibco) supplemented with 10% (v/v) FBS (Gibco) 1× penicillin (Gibco) and 2 mM l-glutamine (Gibco). TRAIP-wt and TRAIP-3A cells were grown in the presence of 500 μg/ml of G418. All cells were passaged when they reached 80% confluence.

### Western blotting

To detect endogenous and TRAIP-wt/-3A protein levels, cells were grown in 6-well plates. For treated samples, medium was supplemented with 500 μM auxin (IAA). For whole cell extract (WCE) preparation, the cell pellet was lysed on ice for 30 min in RIPA Buffer (10 mM Tris–HCl, pH 8.0, 1 mM EDTA, 0.5 mM EGTA, 1% Triton X-100, 0.1% sodium deoxycholate, 0.1% SDS, 150 mM NaCl) with protease inhibitors (Thermo Fisher). WCE was sonicated using the Bioruptor (Diagenode) on high for 10 min, 30 s on × 30 s off at 4°C. Samples were then cleared by centrifugation at 14 000 × *g* for 10 min and prepared for loading by adding 2× SDS Laemmli Buffer (Bio-Rad), supplemented with β-mercaptoethanol (BME), and boiled at 95°C for 5 min. Samples were run on a 4%–12% gradient SDS–PAGE gel (Invitrogen) and transferred to PVDF membranes for 90 min at 100 V. After transfer, membranes were blocked in 5% milk in TBST for 1 h and incubated overnight at 4°C in primary antibody. Membranes were then washed in TBST and incubated in secondary antibody for 1 h at RT. The membranes were again washed in TBST before being developed using ECL detection spray (Advantsa WesternBright).

### Colony formation assay

For colony formation assay, 1000 cells were seeded in six-well plates, with three technical replicates per condition in each experimental repetition. Twenty-four hours later, auxin and/or aphidicolin in different combinations were added to wells with final concentrations of 500 μM and 50 nM, respectively. Cells were grown for another 10 days to form colonies. At this point, cell medium was removed, and colonies were stained with 2% methylene blue in 50% ethanol staining for 20 min at RT. Finally, excess stain was removed by washing and colonies were counted using Oxford Optronix Gel Count plate reader and Gel Count software. Colony formation efficiencies for each cell line and condition (± aphidicolin) were calculated by normalizing number of colonies counted for each cell line grown in the presence of auxin to number of colonies grown without auxin in the same condition and plotted on a graph.

### MiDAS

Cells seeded into six-well plates were arrested in G2 phase of cell cycle by treatment with 9 μM CDK1 inhibitor (RO-3306, Adooq Bioscience) in the presence of 400 nM aphidicolin (Bio-Techne Limited) for 16 h. Ninety minutes before release, cells were treated with DMSO or 500 μM IAA (auxin), and released from G2 arrest by washing them in warm PBS three times and released in fresh medium with 10 μM EdU supplemented with auxin or DMSO for 30 min. Subsequently, cells were washed in PBS, trypsinized, and collected by centrifugation at 500 × *g* for 5 min. The cell pellet was resuspended in 70% ethanol and stored at −20°C until staining. Cells were washed twice in 1 ml 1% bovine serum albumin in PBS and then the pellet was carefully resuspended in Click-iT-AlexaFluor 647 reaction mix (Thermo Fisher) as per manufacturer’s instructions and incubated in dark for 30 min. Finally, cells were cyto-spinned on glass cover slips at 500 × *g* for 8 min and nuclei were stained by DAPI mounting medium. All images were acquired with a Nikon Eclipse E600 microscope with 100× oil objective. For each sample, ≥100 mitotic cells were counted for a set of samples.

### Flow cytometry

Asynchronous cells seeded in six-well plates were treated with auxin or DMSO overnight, and then pulsed with 10 μM EdU for 20 min. Then, cells were washed in PBS, trypsinized, and collected by centrifugation at 500 × *g* for 5 min. The cell pellet was resuspended in 70% ethanol and stored at −20°C until staining, using EdU click-it as above, and DNA staining with Vybrant DyeCycle stains (Invitrogen). Cells were analysed using Beckman Coulter CytoFLEX S. Cell profiles were obtained with CytExpert Software for CytoFLEX, with G1, S, and G2/M cells gated according to DNA content and EdU incorporation.

### Statistical analysis

Student’s *t*-test or one-way ANOVA was used to compare the differences between two or more groups. All statistical calculations were performed using the software Prism (GraphPad). *P*-values <.05 were considered statistically significant.

## Results

### TRAIP interacts with terminated replisomes in S-phase

**Figure 1.**
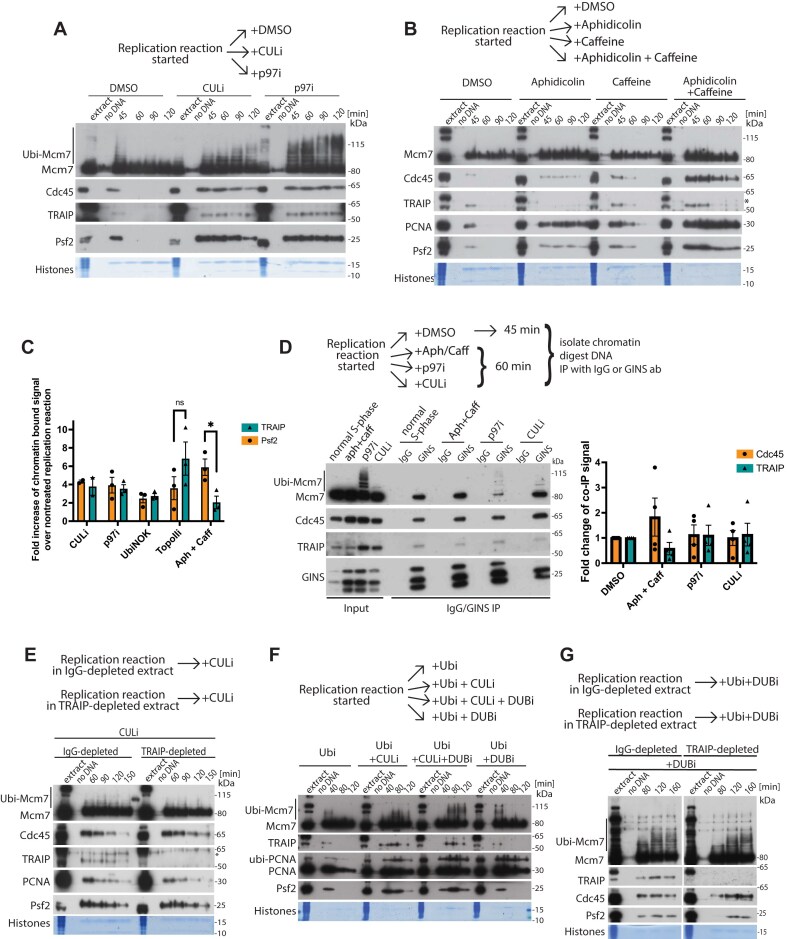
TRAIP interacts with terminated replisomes in S-phase but does not drive their disassembly. (**A**) DNA replication was set up in *Xenopus* egg extract with optional addition of CULi (MLN4924) or p97i (NMS873) and at the indicated times chromatin was isolated and chromatin-bound factors resolved on SDS–PAGE and immunoblotted with indicated antibodies. ‘No DNA’ control served as chromatin specificity control, while Coomassie-stained histones from the bottom of the gel served as a loading and purity control. Representative experiment out of *n* = 3. Quantification in panel (C). (**B**) Replication reaction was set up in egg extract in the optional presence of aphidicolin, caffeine, or both. Chromatin fractions were analysed by immunoblotting as in panel (A). ^*^indicates non-specific band. Quantification from *n* = 3 experiments presented in Supplementary Fig. S1C. (**C**) Quantification of fold enrichment of TRAIP and Psf2 chromatin-bound signals in different treatments over the DMSO control in *n* = 3 experiments. Mean with SEM as error bars is presented. Unpaired *t*-test: *P*= .0269. (**D**) Chromatin replication reaction was carried out in the presence of caffeine and indicated inhibitors. Chromatin was isolated after 45 min of replication reaction for normal S-phase control and 60 min for inhibitor treatments; protein complexes released from chromatin by digestion of DNA with benzonase and non-specific control antibodies or α-GINS antibodies were used for immunoprecipitation (IP). The input and immunoprecipitated samples were analysed by immunoblotting with indicated antibodies. Quantification of TRAIP signal from *n* = 4 experiments is presented. Mean with SEM as error bars is presented. (**E**) DNA replication was set up in IgG- or TRAIP-depleted egg extracts supplemented with CULi, and at the indicated times, chromatin was isolated and chromatin-bound factors were resolved on SDS–PAGE and immunoblotted with indicated antibodies (*n* = 2). ‘No DNA’ control served as chromatin specificity control, while Coomassie-stained histones from the bottom of the gel served as a loading and purity control. ^*^indicates non-specific band. (**F**) Replication reaction was set up in egg extract in the presence of CULi and optionally DUBi (UbiVs). Chromatin fractions isolated at indicated times were analysed by immunoblotting as in panel (A) (*n* = 3). (**G**) DNA replication was set up in IgG- or TRAIP-depleted egg extracts supplemented with CULi and DUBi. Chromatin fractions were analysed by immunoblotting as in panel (A) (*n* = 2).

Our first aim was to understand whether TRAIP interaction with the substrate, the replisome, was regulated during S-phase. To this end, we used cell-free *Xenopus laevis* egg extract system that can efficiently replicate chromatin DNA under regulation remarkably similar to other higher eukaryotic cellular systems [28]. First, we analysed chromatin binding of TRAIP under different conditions known to accumulate terminated or stalled replisomes on chromatin. When analysing replisomes on chromatin, Mcm2–7 complexes are not completely unloaded from chromatin during replication progression due to huge excess of licensed origins on *Xenopus* chromatin [6]. For that reason, to follow active replisomes we focus on Cdc45 and GINS chromatin binding. As seen previously, we could detect TRAIP interacting with chromatin during unperturbed conditions, at the same time as other replication factors (Fig. 1A–C and Supplementary Fig. S1A and C) [9]. Moreover, upon inhibition of replisome disassembly, either through inhibition of Cul2^LRR1^ (inhibition of activatory neddylation of cullins, CULi) or inhibition of p97 segregase activity (p97i) or inhibition of polyubiquitylation with chain-terminating mutant of ubiquitin (UbiNOK), TRAIP accumulated on chromatin together with other replisome components (Fig. 1A and Supplementary Fig. S1A). This was also observed when replisomes were accumulated on chromatin due to inhibition of termination with Topo2 inhibitor ICRF-193 (Supplementary Fig. S1B). However, TRAIP interaction with replisomes is not obligatory during S-phase and is indeed regulated, as we could detect inhibition of TRAIP chromatin binding when replicating extracts were treated with aphidicolin, an inhibitor of replicative polymerases (Fig. 1B and quantified in Supplementary Fig. S1C). The comparison of TRAIP accumulation on chromatin in all these conditions of replisome enrichment indicates that aphidicolin treatment reduces TRAIP chromatin binding (Fig 1C and Supplementary Fig. S1C). We next immunoprecipitated replisomes (GINS IP) from chromatin replicating in the optional presence of some of these inhibitors, and confirmed that TRAIP indeed interacts with GINS and the replisomes when bound to the chromatin (Fig. 1D). Altogether, TRAIP is interacting with terminated replisomes or replisomes stalled by torsional stress just before fork convergence during termination. However, TRAIP’s interaction with replisomes during S-phase is not obligatory as it is regulated and disrupted upon polymerase–helicase decoupling.

### TRAIP does not contribute to unloading of terminated replisomes in S-phase

Our next question was therefore whether TRAIP can contribute to ubiquitylation of Mcm7 and unloading of the terminated replisomes in S-phase. TRAIP was shown to be dispensable for replisome unloading during S-phase when Cul2^LRR1^ unloads terminated replisomes in the *Xenopus* egg extract system, *Caenorhabditis elegans* embryos, and human cells [9, 17, 22] (Supplementary Fig. S1D). We tested therefore whether it can facilitate replisome unloading in S-phase when Cul2^LRR1^ is inhibited for a prolonged time, as we usually observe slow ubiquitylation of Mcm7 and slow unloading of replisomes upon CULi treatment (Fig. 1A and E). However, immunodepletion of TRAIP from egg extract (Supplementary Fig. S1D) does not inhibit the slow unloading of terminated replisomes from chromatin upon cullin inhibition (Fig. 1E), suggesting that it is most likely the incomplete inhibition of Cul2^LRR1^, rather than TRAIP, that is responsible for this effect.

Next, we wanted to emulate the potentially harmful overexpression of TRAIP [18] to see whether this could lead to increased Mcm7 ubiquitylation and unloading of terminated replisomes in S-phase. Addition of high concentration of neither wt TRAIP (TRAIP-wt) nor E3 RING domain enzymatic activity mutant of TRAIP (TRAIP-RING) [10] affects normal S-phase progression or replication (Supplementary Fig. S1E). In conditions of Cul2^LRR1^ inhibition, additional TRAIP-wt led to a modest increase in Mcm7 ubiquitylation and did not stimulate replisome unloading (Supplementary Fig. S1F). Similarly, a modest increase in ubiquitylated Mcm7 was also observed when we additionally inhibited p97 to retain ubiquitylated proteins on chromatin (Supplementary Fig. S1G). Altogether, these results suggest that despite its robust interaction with terminated replisomes in S-phase, TRAIP does not contribute to their ubiquitylation and unloading.

The possibility exists, however, that TRAIP is active, but its ubiquitylation is counteracted by efficient deubiquitylating enzymes (DUBs) under these conditions. To test this, we used UbiVS, an efficient inhibitor of DUBs (DUBi). As inhibiting DUBs reduces the pool of free ubiquitin in the extract, it is essential to add free ubiquitin into the system to avoid the inhibition of polyubiquitylation [5] (Supplementary Fig. S1H). When we simultaneously inhibit Cul2^LRR1^ and DUBs, indeed we can observe clear increase in length of chains built on Mcm7, suggesting that DUBs are acting on these chains (Fig. 1F). We therefore tested whether TRAIP is responsible for this ubiquitylation counteracted by DUBs by analysing Mcm7 ubiquitylation in TRAIP-depleted extract treated with CULi and DUBi (Fig. 1G). We observed very small differences between IgG- and TRAIP-depleted extracts, again suggesting that most of the observed Mcm7 ubiquitylation is the result of not completely inhibited Cul2^LRR1^.

Altogether, our data suggest that despite interacting with the substrate, the terminated replisomes, TRAIP does not contribute much to ubiquitylation of Mcm7 in S-phase. Finally, the length of TRAIP-dependent ubiquitin chains that we observe in mitosis decorating Mcm7 is always much longer (Supplementary Fig. S1I), suggesting that TRAIP is specifically activated in mitosis to deliver this efficient ubiquitylation of Mcm7. This could be due to novel interaction partners, post-translational modifications, or absence of factors that could inhibit TRAIP activity in S-phase.

### TRAIP is phosphorylated in mitosis

**Figure 2.**
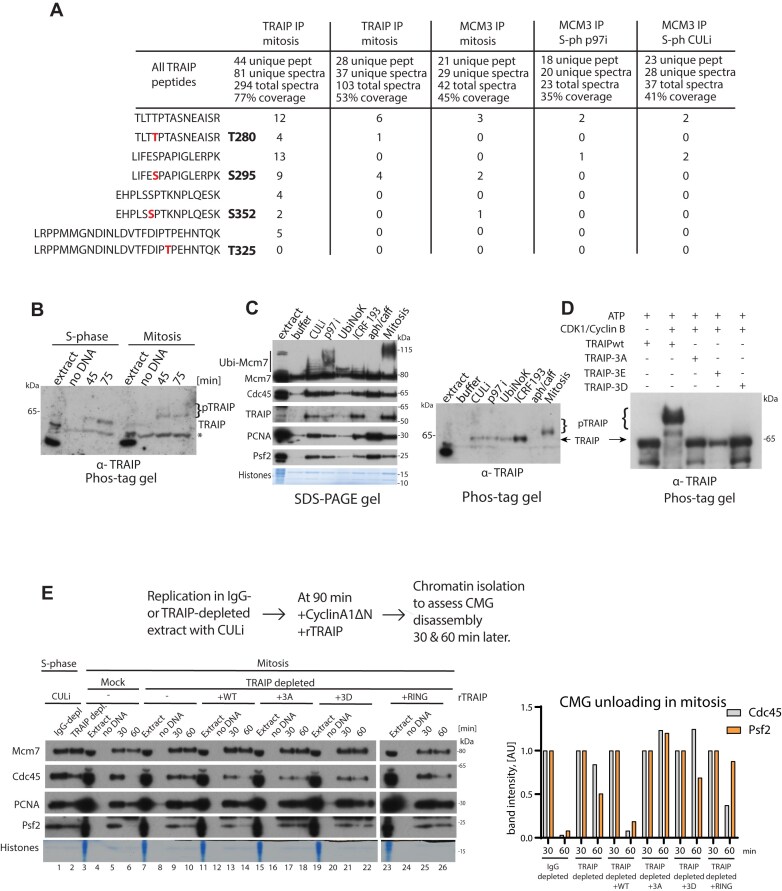
TRAIP is phosphorylated in mitosis by CDKs and this phosphorylation is essential for replisome unloading in mitosis. (**A**) Samples of Mcm3- IPs from chromatin either in S-phase or in mitosis were analysed by mass spectrometry, as previously reported [4, 10]. Additionally, two IPs of TRAIP (different antibodies) from mitotic chromatin were analysed. Number of TRAIP trypsin digestion peptides containing identified phosphorylation sites either in phosphorylated or in non-phosphorylated form are reported. The number of TRAIP unique peptides, unique spectra (includes peptides with differential modifications), total spectra, and protein coverage are also presented for comparison between experiments. Only peptides with >90% confidence of phosphorylation identification are reported. The data for the peptide containing the fourth putative CDK phosphorylation site are also presented. (**B**) Chromatin samples were isolated either from S-phase or from mitosis (TRAIP retained due to p97i) at indicated times, separated on phos-tag gel, and TRAIP visualized by western blotting with TRAIP antibodies (*n* = 4). * indicates non-specific band. (**C**) Replication reaction was set up in egg extract in the optional presence of indicated inhibitors; chromatin samples isolated at 60 min of reaction or upon induction of mitosis with CyclinA1ΔN + p97i. Chromatin samples were separated on standard SDS–PAGE (4%–12% gradient gel) or on phos-tag gel (*n* = 3). Indicated proteins were analysed by western blotting and results are shown as in Fig. 1. (**D**) Purified *X. laevis* His6-SUMO-TRAIP-wt or indicated mutants were *in vitro* phosphorylated with CDK1/Cyclin B, separated on phos-tag gel, and visualized by immunoblotting with TRAIP antibodies (*n* = 6). (**E**) Replication reaction was set up in IgG- or TRAIP-depleted extracts in the presence of CULi. After completion of DNA synthesis (90 min), CyclinA1ΔN was added to the extracts to drive them into mitosis. At the same time, recombinant TRAIP-wt or indicated mutants were added at 1 μM concentration. Chromatin was isolated at indicated times after CyclinA1ΔN addition to assess CMG disassembly. The proportion of Cdc45 and Psf2 present on chromatin at 60 min in comparison to 30 min was quantified in this experiment. Quantification of Cdc45 in an analogous independent experiment is presented in Supplementary Fig. S2E.

First, we were interested in analysing the composition of terminated replisomes in S-phase or after transition to mitosis. To this end, we re-analysed our previously published mass spectrometry data of Mcm3 IP from chromatin with replisomes retained on chromatin at the end of S-phase due to inhibition of Cullin and p97 activity (from [4]) and Mcm3 IP from mitotic chromatin with replisomes retained due to p97 inhibition (from [10]). We compared the most abundant interactors (Supplementary Table S1) and found that mitotic replisomes were enriched in a number of factors, including (i) histone H1, which was shown to play an important role in the structure and function of *X. laevis* chromosomes in mitosis [41], (ii) FancD2 and Rad52 that were shown to be key components of MiDAS response [42, 43], (iii) WRN helicase, which protects from aberrant mitotic recombination [44], and (iv) KIF1—a microtubule motor protein acting in mitosis. At the same time, replisomes in mitosis show a depletion of a number of important factors such as Mcm10, Ctf18, Claspin, Parp1, and Donson. Altogether, the architecture of replisomes retained on chromatin in S-phase and in mitosis is quite different. Interestingly, the re-analysed data of Mcm3 IPs from S-phase or mitosis found that uniquely in mitosis the replisome-bound TRAIP was phosphorylated (Fig. 2A). This was confirmed through precipitation of TRAIP from mitotic chromatin with two independent antibodies. Altogether, we found TRAIP phosphorylated on mitotic chromatin at three sites (T280, S295, and S352). All three mapped sites were S/T-P motifs; this suggested that they are likely to be phosphorylated by CDKs. There is a fourth potential CDK phosphorylation site (T325) within TRAIP, but it is located within a 27-aa-long peptide, which presents a problem for mass spectrometry detection. Therefore, only in one of our TRAIP IPs we could identify only five peptides containing T325 and none of them was phosphorylated. Nevertheless, all four of these phosphorylation sites show good conservation through evolution either directly or in close proximity (Supplementary Fig. S2A).

To confirm that we can detect TRAIP phosphorylation on mitotic chromatin using a different approach, we run chromatin samples from S-phase and mitosis on phos-tag gel and could observe gel retardation of phosphorylated TRAIP protein bound to chromatin in mitosis (Fig. 2B). We then replicated DNA under different conditions that accumulate TRAIP on chromatin in S-phase (Fig. 1), isolated chromatin from these reactions, and run them on normal or phos-tag gel alongside chromatin sample isolated from mitosis (Fig. 2C). We could again observe the superior level of Mcm7 ubiquitylation in mitosis chromatin sample, and this was the only sample in which we could observe phosphorylation shift of TRAIP on phos-tag gel. Moreover, as all of the TRAIP is running at higher molecular weight, this suggests that most, if not all, of chromatin-bound TRAIP in mitosis is phosphorylated (Fig. 2C).

As all three mapped sites were likely to be CDK phosphorylation sites, we tested that recombinant TRAIP can be phosphorylated *in vitro* by CDKs. CDK1–CyclinB could indeed phosphorylate efficiently purified TRAIP, and mutation of the three identified phosphorylation sites (TRAIP-3A) completely abolished this phosphorylation (Fig. 2D and Supplementary Fig. S2B), suggesting that either T325 is not phosphorylated by CDKs or the phosphorylation of all of these sites is cooperative and mutation of the remaining three sites abolishes the phosphorylation of the fourth. Comparing different CDKs–cyclin combinations at equimolar concentrations revealed that the mitotic CDK1–CyclinB and CDK2–CyclinA, which drive mitosis in *Xenopus* embryos, are both able to phosphorylate TRAIP [45] (Supplementary Fig. S2C).

### Phosphorylation of TRAIP is essential for terminated replisome unloading in mitosis

The recombinant TRAIP with all three phosphorylation sites mutated (TRAIP-3A) cannot be phosphorylated by CDKs *in vitro* (Fig. 2D). We have wondered therefore whether such a mutant will be able to ubiquitylate Mcm7 and drive replisome unloading in mitosis. To this end, we run replication reactions in IgG- or TRAIP-depleted egg extract in the presence of CULi, and upon completion of DNA replication added CyclinAΔN to drive extract into mitosis as before [10]. At the same time, we optionally supplemented extract with TRAIP-wt, TRAIP-RING (catalytically dead), TRAIP-3A, or TRAIP-3D. We isolated chromatin samples and analysed Mcm7 ubiquitylation and replisome unloading under these conditions (Fig. 2E). As expected, TRAIP-depleted extract was unable to unload terminated replisomes in mitosis (Fig. 2E, lane 10 versus lane 6), and addition of TRAIP-wt (Supplementary Fig. S2D) could rescue this (Fig. 2E, lane 14), but not addition of catalytically dead RING mutant (TRAIP-RING) (Fig. 2E, lane 26). Importantly, supplementing extract with TRAIP-3A, which cannot be phosphorylated, reproducibly cannot rescue terminated replisomes unloading in mitosis (see Fig. 2E, lane 18, and Supplementary Fig S2E for Cdc45 quantification in an analogous independent experiment). We could also observe a delay in terminated replisome unloading in mitosis when TRAIP-3A was supplemented at high concentration in normal egg extract to compete with endogenous TRAIP (imitating mutant overexpression conditions in cells) (Supplementary Fig. S2F). We have also tested a potential phospho-mimicking mutant of TRAIP where all three residues were mutated to negatively charged aspartic acid (TRAIP-3D; Supplementary Fig. S2B and Fig. 2E). However, the presence of charged amino acids could not support TRAIP activity in mitosis, with TRAIP-3D acting analogously to TRAIP-3A (Fig. 2E). Altogether, we propose that phosphorylation of TRAIP at T280, S295, and S352 is essential for mitotic activity of TRAIP.

### Phosphorylation of TRAIP stimulates its ubiquitin ligase activity

We wanted next to understand how phosphorylation of TRAIP could affect its activity in mitosis. TRAIP has been suggested to form a dimer as its coiled coil domain can dimerize [46]. AlphaFold3 [34, 47] simulation of TRAIP structure indeed predicts that the central coiled coil domains of TRAIP dimerize, with two N-terminal RING E3 domains coming together, a flexible hinge in the middle of the folded molecule, and a disordered C-terminus (Supplementary Fig. S3A). Our XL-MS analysis of purified recombinant *X. laevis* His-SUMO-TRAIP revealed a number of cross-links that could be possibly formed between two molecules of TRAIP or within the same molecule. However, two of them could only be formed if TRAIP formed a dimer (Supplementary Fig. S3B), strongly supporting the notion that TRAIP exists as a homodimer. Due to its elongated and flexible structure, purified recombinant TRAIP migrates in gel filtration at a position of much larger complexes (Supplementary Fig. S3C–E). However, it migrates through the glycerol gradient at a smaller molecular size (Supplementary Fig. S3F). We used the Siegel–Monty method [33] to combine the Stoke’s radius and sedimentation coefficient for TRAIP and calculate its apparent molecular size, which at 138 kDa corresponds well to a dimer of two SUMO-TRAIP molecules at 65 kDa each.

All three of the TRAIP phosphorylation sites are located within the unstructured flexible C-terminal part of TRAIP (Supplementary Fig. S3A). CDK-driven phosphorylation of TRAIP is not changing TRAIP’s migration pattern in the gel filtration, arguing against drastic conformational changes induced by phosphorylation (Supplementary Fig. S3D and E). However, AlphaFold3 predicts that when the three mapped residues are phosphorylated, the unstructured C-terminus can be reorganized without affecting the dimer TRAIP formation (Supplementary Fig. S3G). Such a reorganization has not been predicted for single phosphorylations, nor mutations to the aspartic or glutamic acids (Supplementary Fig. S3G and data not shown). We have also performed parallel XL-MS of non-phosphorylated and phosphorylated SUMO-TRAIP (Supplementary Fig. S3H). Interestingly, we can observe a different pattern of cross-links in the disordered C-terminal region of the protein when comparing phosphorylated and non-phosphorylated TRAIP. While non-phosphorylated TRAIP shows seven cross-links, phosphorylated TRAIP only two, of which one is in common (lysine: 387–394). The presence of cross-links between long distanced residues in non-phosphorylated TRAIP (lysine: 355–238, 270, 437–442) indicates that these regions are/can be topologically close to each other. Moreover, we detect fewer monolinks (free cross-linker attachments) in the first part of the disordered region in phosphorylated TRAIP. These results can suggest a possible change of conformation upon phosphorylation to a more compact structure within the disordered region (reduction of accessibility of lysines), but can also be influenced by possible impact of phosphorylation on detection of cross-linked peptides (Supplementary Fig. S3H and I). This possibility of conformational change led us to hypothesize that phosphorylation of TRAIP could affect its ubiquitin ligase activity, which could explain differential activity towards the substrate, the CMG helicase.

**Figure 3.**
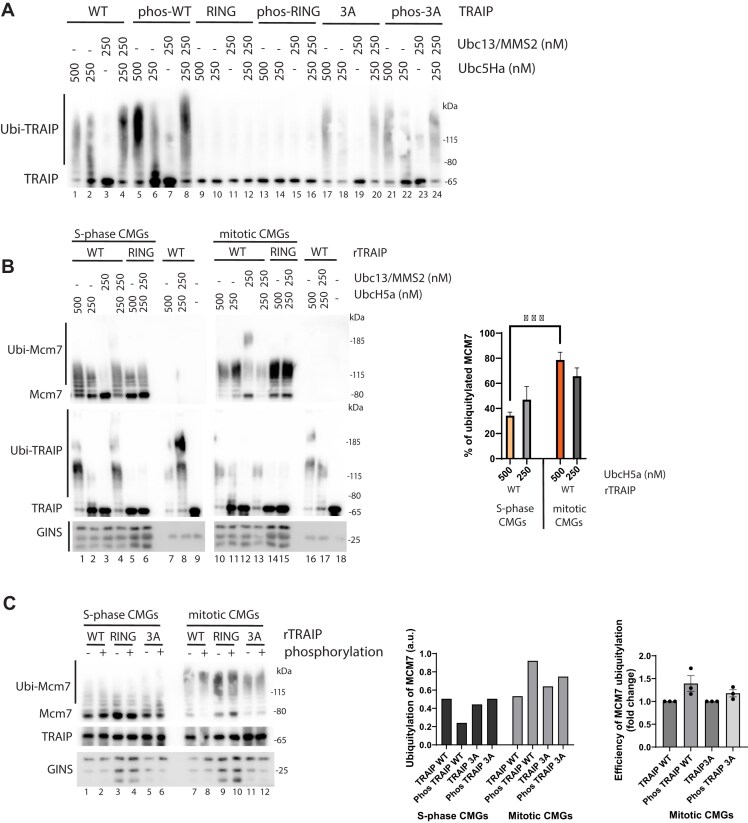
Phosphorylation of TRAIP increases TRAIP’s E3 ligase activity. (**A**) *In vitro* ubiquitylation reaction was set up in the presence of ubiquitin, E1, ATP, indicated concentrations of E2 enzymes, and indicated mutants of His6-SUMO-TRAIP. The mutants of His6-SUMO-TRAIP were also optionally phosphorylated with CDK1/Cyclin B before the ubiquitylation reaction. Samples from each ubiquitylation reaction were run on SDS–PAGE and immunoblotted with α-TRAIP antibodies to assess TRAIP autoubiquitylation (*n* = 3). (**B**) Post-termination CMGs were isolated from S-phase (CULi and p97i) or mitotic chromatin (CULi, p97i, and CyclinA1ΔN) and used as a substrate for *in vitro* ubiquitylation by TRAIP as in panel (A). The ubiquitylation products were analysed with α-TRAIP and α-Mcm7 antibodies, while α-GINS blot serves as a loading control. Reactions without CMGs were run in parallel to ensure specificity of Mcm7 signal, while the reactions with TRAIP-RING mutants show the level of carried over ubiquitylation present on Mcm7 isolated from the chromatin. The level of *in vitro* ubiquitylation of Mcm7 in S-phase and mitotic CMGs was calculated by quantification of basic forms of non-ubiquitylated Mcm7 in each lane and compared to the signal of non-ubiquitylated Mcm7 in TRAIP-RING lanes. Mean values are presented with SD, *n* = 3, two-tailed Student’s *t*-test, ***P-value <.001. (**C**) The S-phase and mitotic CMGs were ubiquitylated in the presence of 250 nM UbcH5 by optionally phosphorylated His6-SUMO-TRAIP prior to ubiquitylation reaction. Ubiquitylation of Mcm7 within CMGs in this experiment was analysed as in panel (B). The level of ubiquitylation of Mcm7 in mitotic replisomes in *n* = 3 experiments was quantified. Mean values are presented with SEM.

TRAIP has been shown previously to be efficient at autoubiquitylation *in vitro* in combination with UbcH5 as E2 conjugating enzyme [48], but we have identified Ubc13-Mms2 (Ube2V1-Ube2n) E2 conjugating enzyme accumulating on mitotic chromatin with retained post-termination replisomes [10]. We have therefore tested autoubiquitylation of TRAIP with both E2 enzymes. Unlike Ubc13-Mms2, UbcH5 could support autoubiquitylation of TRAIP on its own (Supplementary Fig. S3J); however, the most efficient combination was equimolar mix of both E2s at concentration that allowed monoubiquitylation by UbcH5 and extension of the chain by Ubc13-Mms2 (Supplementary Fig. S3K). All TRAIP phosphorylation mutants (TRAIP-3A, TRAIP-3D, and TRAIP-3E) could support autoubiquitylation of themselves (Supplementary Fig. S3L). With this set-up, we tested whether CDK phosphorylation affects TRAIP autoubiquitylation activity. We could observe that phosphorylated TRAIP was much more decorated with ubiquitylation chains in all E2 combinations (Fig. 3A, lanes 5–8 versus lanes 1–4) and that this was TRAIP E3-ligase dependent as TRAIP-RING mutant showed no activity (Fig. 3A, lanes 9–16). Moreover, TRAIP-3A mutant was not stimulated by CDK phosphorylation (Fig. 3A, lanes 17–24) arguing against the possibility that CDK phosphorylation stimulates any other enzymes in the ubiquitylation reaction. This suggests that, indeed, phosphorylation of TRAIP introduces conformational changes within the protein that can affect its ubiquitin ligase activity.

We next tested whether similar stimulation can be observed with TRAIP ubiquitylation of the substrate—the replisome. To this end, we supplemented replication reaction assembled in the egg extract with *X. laevis* rCdc45-FLAG, CULi, and p97i. At 90 min, when DNA synthesis was completed and most of accumulated replisomes were in terminated form, we either isolated chromatin or supplemented the reaction with CyclinAΔN to drive extract into mitosis, and then we isolated chromatin 30 min after CyclinAΔN addition. The terminated replisomes present on chromatin either in S-phase or in mitosis were released from chromatin by benzonase digestion and purified through FLAG-IP and elution with the FLAG-peptide as previously described [49]. The isolated terminated S-phase or mitotic replisomes were used as a material for *in vitro* TRAIP ubiquitylation (Fig. 3B). The isolated CMGs contain Mcm7 that has been ubiquitylated in the extract—the level of this endogenous ubiquitylation is visible in reactions containing TRAIP-RING, which is unable to provide further *in vitro* ubiquitylation (Fig. 3B, lanes 5–6 and 14–15). In the presence of only Ubc13/MMS2, the endogenous ubiquitin chains attached to Mcm7 are extended by TRAIP/Ubc13/MMS2 and they become very long or not even able to enter the gel (lanes 3 and 12). However, the level of unmodified Mcm7 in these samples is comparable to isolated level of Mcm7 visible in TRAIP-RING samples, suggesting that there is no *de novo* priming ubiquitylation of Mcm7 (lanes 3 and 12). In the presence of UbcH5, we can observe more *de novo in vitro* ubiquitylation of Mcm7 (lanes 1–2 and 10–11). We observed that recombinant TRAIP can ubiquitylate Mcm7 within mitotic replisomes more efficiently than S-phase replisomes, with much less unmodified Mcm7 retained in mitotic samples (Fig. 3B). To determine the effect of phosphorylation of TRAIP on its activity towards S-phase and mitotic replisomes, we chose to analyse *in vitro* ubiquitylation of CMGs in the presence of only 250 nM UbcH5, as this reaction showed room for improvement, with not all of Mcm7 ubiquitylated with non-phosphorylated TRAIP (Fig. 3B). We have therefore optionally phosphorylated TRAIP and its mutants with CDK1/Cyclin B and observed that phosphorylated TRAIP is more efficient at ubiquitylating Mcm7 within the mitotic replisomes (Fig. 3C, lane 7 versus lane 8). No such stimulation was observed upon phosphorylation of TRAIP-3A or TRAIP-RING mutants (Fig. 3C, lanes 9–12). Again, the ubiquitylation activity and stimulation by phosphorylation were observed only for mitotic, and not S-phase, replisomes.

Altogether, our results indicate that CDK-driven mitotic phosphorylation of TRAIP is essential for its ability to unload replisomes retained on chromatin until mitosis in *Xenopus* egg extract. This phosphorylation does not change significantly TRAIP dimer conformation but stimulates autoubiquitylation activity of TRAIP and to a lesser extent its activity towards mitotic replisomes.

### Phosphorylation of TRAIP is important for mitotic activities of TRAIP in human cells

**Figure 4.**
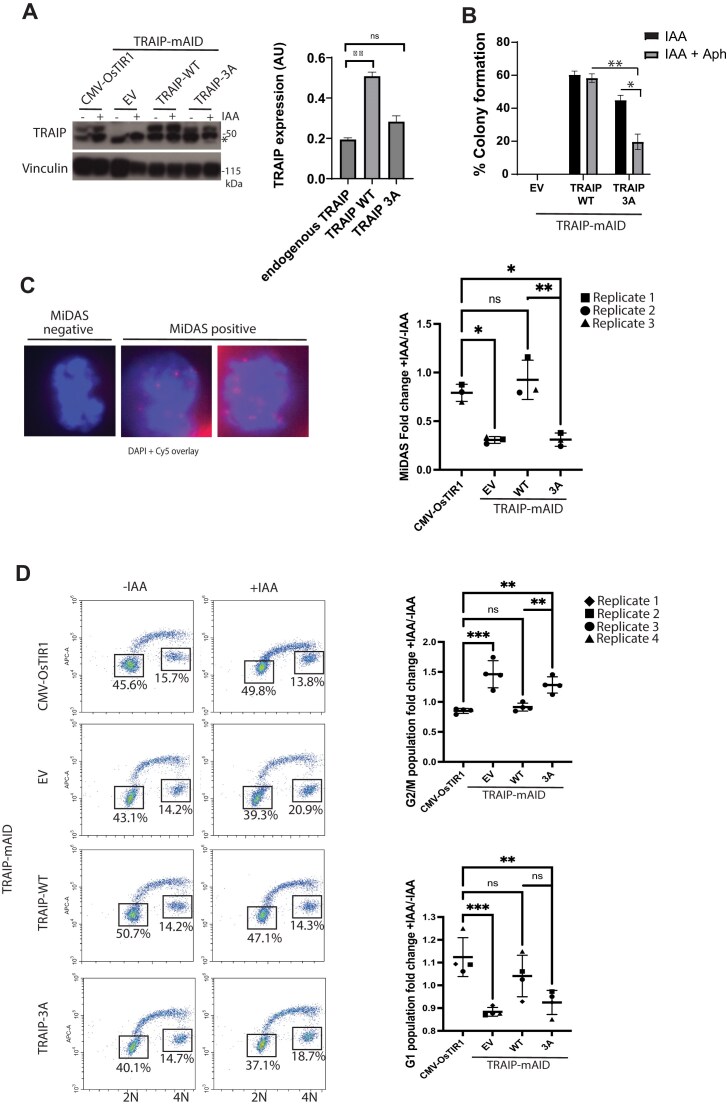
TRAIP phosphorylation is essential for MiDAS in human cells. (**A**) The expression levels of endogenous TRAIP, TRAIP-wt, and TRAIP-3A were analysed by immunoblotting in whole cell extracts (WCE) using α-TRAIP antibody. Bands intensities were quantified and normalized against vinculin as a loading control, based on three independent experiments (*n* = 3). The results are presented as the mean with SEM and two-tailed Student’s *t*-test with **P-value <.005. (**B**) One thousand cells were plated in triplicate, and culture medium was optionally supplemented with 500 μM IAA and 50 nM aphidicolin. Cells were grown until colony formation. Colony formation efficiency normalized to the corresponding untreated sample for each cell line was measured (*n* = 3), mean value presented with SEM, two-tailed Student’s *t*-test, *P-value <.05, **P-value <.01. (**C**) Cells were synchronized in G2 stage of the cell cycle with CDK1 inhibitor RO-3306 in the presence of 400 nM aphidicolin, and then allowed to proceed in G2/M phase of the cell cycle in the presence or absence of 500 μM IAA to degrade TRAIP. DNA synthesis was labelled by 30 min EdU incorporation. DNA synthesis in mitosis (MiDAS) was assessed by microscopy. Representative images of MiDAS-positive and MiDAS-negative cells, DAPI and Cy5 (EdU) overlay images. MiDAS quantification as fold change + IAA/IAA in the corresponding samples (*n* = 3), two-way ANOVA. The number of MiDAS foci per cell presented in Supplementary Fig. S4C. (**D**) Asynchronous cells were treated with IAA overnight, DNA synthesis was labelled with EdU, and fixed cells were stained and analysed by FACS. Representative FACS image shows the population of cells in G1 and G2/M quantified. The graphs show the fold change between samples treated with IAA (+ IAA) and without (− IAA), *n* = 4, two-way ANOVA. The graphs show mean with SEM, *P-value <.05, **P-value <.01, ***P-value <.001.

Having established the essential role for TRAIP phosphorylation in the *Xenopus* model system, we wanted to investigate whether the regulation of TRAIP activity through CDK phosphorylation is equally important in human somatic cells. The human TRAIP protein contains three CDK phosphorylation sites in the same part as in *Xenopus* protein, either directly or closely conserved (Supplementary Fig. S2A). We have therefore generated human TRAIP-3A mutant (S295A, T324A, S347A) for retroviral expression and used previously characterized HCT116 auxin-inducible degron TRAIP-mAID cell line to create cells in which we can constitutively express TRAIP-wt or our mutant and degrade endogenous TRAIP-mAID [17]. First, we established that our proteins expressed at a level similar to endogenous TRAIP, and were not affected by the presence of auxin (IAA) (Fig. 4A and Supplementary Fig. S4A). Next, we assessed cell viability upon TRAIP-mAID degradation by colony formation assay. TRAIP-mAID cells optionally expressing TRAIP-wt or TRAIP-3A were plated with or without auxin and the number of colonies formed with auxin addition was normalized to no-auxin control (Fig. 4B and Supplementary Fig. S4B). We were not able to visualize by western blot the efficiency of TRAIP-mAID degradation in our cells, due to a non-specific band of the antibody used to visualize endogenous levels of TRAIP (Supplementary Fig. S4A). However, addition of auxin completely abolished the proliferation of TRAIP-mAID cells expressing empty vector (Fig. 4B, EV), indicating that in our conditions TRAIP-mAID degradation leads to expected outcomes. Interestingly, we could observe that cells expressing TRAIP-3A as the only form of TRAIP were forming fewer colonies compared to TRAIP-wt, suggesting that these phosphorylation sites are important for full rescue of TRAIP activity, but the effect was small (Fig. 4B and Supplementary Fig. S4B). We therefore treated cells with low level of polymerase inhibitor aphidicolin, which is known to generate under-replication and leads to expression of CFS, both of which are counteracted in mitosis by TRAIP and MiDAS [13, 16]. Upon aphidicolin treatment, cells expressing TRAIP-3A showed significantly lower survival (Fig. 4B and Supplementary Fig. S4B), suggesting that without mitotic phosphorylation, TRAIP cannot function to support cell survival upon replication stress.

As previously mentioned, cells in mitosis respond to replication stress by using MiDAS, and it has been shown that cells depleted of TRAIP are deficient in MiDAS [16]. Therefore, we tested the ability of our cell lines to form MiDAS foci (Supplementary Fig. S4C). To consider the possible effect of auxin itself, we calculated the fold change of auxin treated versus untreated samples (Fig. 4C). Similarly to the colony formation assay data, the expression of TRAIP-3A mutant was not able to rescue MiDAS in TRAIP-mAID cells treated with auxin (Fig. 4C). These data suggest that TRAIP phosphorylation is essential for cell viability and MiDAS formation upon mild replication stress. Finally, it is known that TRAIP degradation arrests cells in G2/M phase of the cell cycle, with accompanying decrease in the number of cells in G1 phase [17]. We analysed the cell cycle profile by FACS in an asynchronous population. We observed a slight effect of auxin addition on its own on G1 and G2/M populations (Supplementary Fig. S4D and E); therefore, as for MiDAS, we calculated the fold change of auxin treated versus untreated. We confirmed that the expression of TRAIP-wt was sufficient to rescue both the G2/M accumulation phenotype and the G1 decrease, while the TRAIP-3A mutant was defective in the rescue (Fig. 4D). Altogether, these observations indicate that the requirement for phosphorylation of TRAIP by CDKs in mitosis is conserved in evolution and required for mitotic functions of TRAIP in human cells.

### CDK phosphorylation of TRAIP is not sufficient for replisome unloading

**Figure 5.**
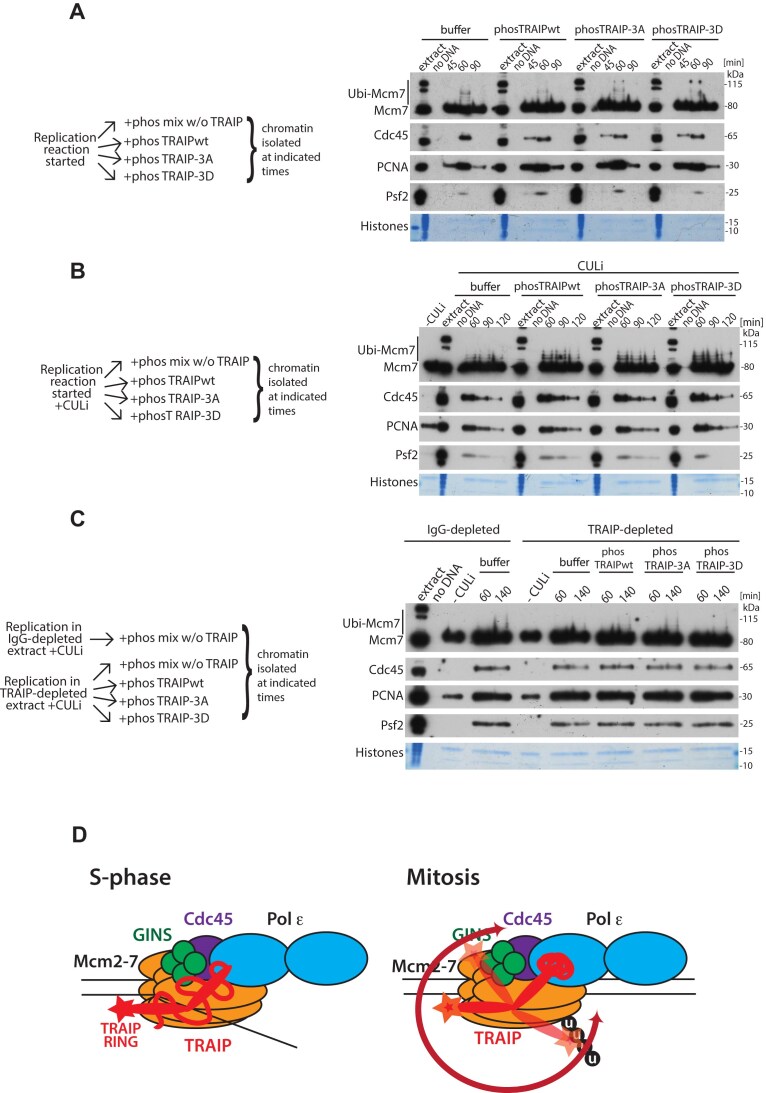
Phosphorylation of TRAIP is not sufficient to induce uncontrolled replisome ubiquitylation in S-phase. (**A**) DNA replication reaction was set up in egg extract and optionally supplemented with 1.5 μM TRAIP, either wt or mutant, phosphorylated in vitro by CDK1/Cyclin B. Chromatin samples were isolated at indicated times and analysed by immunoblotting as in Fig. 1 (*n* = 3). (**B**) As in panel (A), but the replication reaction was additionally supplemented with CULi to assess whether *in vitro* phosphorylated TRAIP can support ubiquitylation and unloading of accumulated terminated replisomes. Chromatin samples were analysed as in panel (A). −CULi sample was isolated at 75 min without TRAIP addition as a control for the inhibitor activity (*n* = 1). (**C**) Replication reactions were set up in IgG- or TRAIP-depleted extracts supplemented with CULi (apart from the indicated −CULi controls; isolated at 75 min) to accumulate terminated replisomes on chromatin. *In vitro* phosphorylated TRAIP (wt or mutant) was added to TRAIP-depleted extract as indicated. Samples were analysed as in Fig. 1 (*n* = 3). (**D**) Model of proposed TRAIP engagement with the replisome in S-phase and in mitosis. In mitosis, the CDK-driven phosphorylation of TRAIP affects its 3D structure, making it more active towards the replisomes, possibly through increased flexibility of TRAIP. There are likely additional changes to the mitotic replisome that facilitate TRAIP activity, as isolated mitotic replisomes are a better substrate for TRAIP activity *in vitro*.

Our data thus far indicate that phosphorylation of TRAIP by mitotic CDKs is essential for its activity and unloading of replisomes in mitosis, both in *X. laevis* egg extract and in human cell lines. We were wondering, therefore, whether phosphorylation of TRAIP by CDKs is sufficient to enable TRAIP ubiquitylation activity towards Mcm7 and unloading of terminated replisomes in S-phase. The phospho-mimicking mutants of TRAIP cannot substitute for phosphorylated TRAIP protein either in *in vitro* ubiquitylation reactions or in mitotic egg extract (Figs 2E and 3A). We therefore decided to *in vitro* phosphorylate recombinant TRAIP with CDK1/Cyclin B and add it to the S-phase replication reaction. Such *in vitro* pre-phosphorylated TRAIP-wt is at least equally efficient, if not slightly better, at unloading of post-termination replisomes in mitosis, when compared to non-pre-phosphorylated TRAIP-wt, which needs to gain its phosphorylation after addition to the mitotic extract (Supplementary Fig. S5A). As a control, we phosphorylated TRAIP-3A or TRAIP-3D, which we have shown do not exhibit any retardation during separation in phos-tag gels, suggesting that they are not phosphorylated (Fig. 2D). Addition of high concentration (5× more than endogenous) of the phosphorylated forms of TRAIP did not affect egg extract ability to synthesize DNA (Supplementary Fig. S5B) during replication reaction, and none of them affected Mcm7 ubiquitylation or replisome unloading (Fig. 5A). We have confirmed that TRAIP remained phosphorylated during the replication reaction (Supplementary Fig. S5C) to avoid the possibility that efficient dephosphorylation is responsible for the lack of observed activity. This result suggests that exogenously phosphorylated TRAIP cannot uncontrollably ubiquitylate and disassemble active CMGs on chromatin.

Next, we wanted to test whether phosphorylated TRAIP will show more activity specifically towards terminated replisomes during S-phase. To this end, we repeated the above experiment but in the presence of CULi to accumulate terminated replisomes on chromatin in S-phase (Fig. 5B). Again, we observed no effect of TRAIP phosphorylation on Mcm7 ubiquitylation and replisome unloading. Finally, to exclude the possibility that endogenous TRAIP is more efficient at interacting with replisomes and affects our results, we supplemented TRAIP-depleted extract with either phosphorylated TRAIP-wt or mutants (Fig. 5C). Once more, we could see no activity of phosphorylated TRAIP towards terminated replisomes. Altogether, this leads us to conclusion that although phosphorylation of TRAIP by mitotic CDKs is essential for TRAIP function in unloading replisomes in mitosis and MiDAS, it is not sufficient to stimulate TRAIP activity towards S-phase replisomes. This agrees with our findings from *in vitro* ubiquitylation reactions, which suggest that S-phase replisomes are not a good substrate for either phosphorylated or non-phosphorylated TRAIP (Fig. 3B and C). Further studies are required to determine whether any of the many factors associating differentially with S-phase and mitotic replisomes is responsible for regulating TRAIP activity towards Mcm7 within the replisomes, or whether mitosis is inducing additional posttranslational modifications of other replisome factors regulating Mcm7 accessibility and ubiquitylation.

## Discussion

Removal of the replicative helicase from chromatin is an active process driven by two ubiquitin ligases Cul2^LRR1^ and TRAIP. Uncontrolled unloading of replicative helicase during S-phase would be disastrous for genome stability and cell viability. Over the last 5 years, we have understood much better the essential regulation of Cul2^LRR1^ ubiquitin ligase, whose binding to substrate (CMG helicase) is restricted to CMG complexes not engaged with fork DNA structure, preventing Cul2^LRR1^ from uncontrolled removal of active replicative helicase from chromatin. However, our understanding of regulation of TRAIP activity is lacking. Here, we have shown that TRAIP activity towards replisomes is not regulated at the level of interaction with the substrate, as it can robustly interact with terminated replisomes and not ubiquitylate them (Fig. 1 and Supplementary Fig. S1A). As TRAIP can ubiquitylate DNA–protein cross-links (DPCs) standing in the way of progressing replisome [50] and can ubiquitylate replisomes converged at the interstrand cross-link (ICL) [22], it is hypothesized that possibly TRAIP can interact with the replisomes in a way that is able to ubiquitylate factors ahead of the fork (*in trans*) but not the host replisome itself (*cis* ubiquitylation). Direct evidence supporting this hypothesis is, however, missing as we do not know how TRAIP is docked on the replisome. The AlphaFold ‘predictome’ [51] suggests that TRAIP can interact with DNA Pol ϵ catalytic subunit, which would agree with our observation that decoupling of helicase and polymerase upon aphidicolin treatment reduces TRAIP interaction with the CMG helicase (Fig. 1B). No interactions of TRAIP with TIMELESS and TIPIN, which are positioned at the utter most forward-facing end of the replisome [24], have been reported thus far. However, the flexible and elongated form of TRAIP dimer could possibly be docked through the DNA Pol ϵ interaction and reach quite a distance ahead. It is also not clear whether the requirement for TRAIP activity in response to various types of DNA damaging agents [18–21] involves TRAIP ubiquitylating Mcm7 within the replisome encountering the damage or ubiquitylating yet another protein as in case of DPC traversing and repair [50]. The future work will need to uncover the structural aspects of TRAIP–replisome interactions, to understand what prevents TRAIP in S-phase from ubiquitylating the host replisome.

Although here we have not explained the regulatory mechanism inhibiting TRAIP activity in S-phase towards active and terminated replisomes, we have discovered the essential activatory phosphorylation of TRAIP by mitotic CDKs. Our mass spectrometry analysis of chromatin- and replisome-bound TRAIP during mitosis revealed phosphorylation at three CDK phosphorylation consensus sites (T280, S295, and S352). It is likely that *Xenopus* TRAIP is phosphorylated also at the fourth CDK site T325, but its detection by mass spectrometry is hindered due to the large size of the trypsin digestion peptide containing T325 (Fig. 2A). T325 is conserved through evolution and present in human TRAIP (Supplementary Fig. S2A). Nevertheless, the phosphorylation of TRAIP by CDKs seems to depend on recognition of a number of sites as mutation of just the three sites, T280, S295, and S352, to alanines abolishes any CDK phosphorylation *in vitro*, and TRAIP-3A is not able to support replisome unloading in mitosis (Fig. 2D and E). Interestingly, while we were preparing this manuscript, both Walter and Labib groups reported in bioRxiv that phosphorylation of TRAIP in mitosis, including at T325, is essential for TRAIP’s function in replisome unloading [52, 53], suggesting that indeed T325 is likely to be also phosphorylated. These two reports provide evidence that phosphorylation of TRAIP at T325 allows it to bind to the N-terminal end of TTF2 ATPase, which in turn interacts with Pol ϵ regulatory subunit (POLE2). As TTF2 is mainly a cytoplasmic protein in cells during interphase, it gains access to chromatin upon nuclear membrane disassembly in mitosis, providing the mitosis specificity for these interactions. These interactions could stabilize an alternative (different than S-phase) interaction with Pol ϵ, which could support ubiquitylation of the host replisome [52, 53].

TTF2 is undoubtedly an important part of the function of TRAIP in mitosis [52, 53]. However, it is likely not to be the only requirement or regulatory mechanism through which TRAIP is activated in mitosis. Our mass spectrometry analysis of replisomes isolated from S-phase and from mitosis detected very similar number of TTF2 peptides interacting with replisomes in both stages of the cell cycle (27 peptides in mitosis and 25 peptides in S-phase), suggesting that in our model system TTF2 interaction with chromatin is not restricted to mitosis. This may be a specificity of nuclei formed in *Xenopus* egg extract, as they are formed around decondensing sperm chromatin shortly before commencement of DNA replication [28]. Nevertheless, addition of *in vitro* phosphorylated TRAIP to S-phase extract does not stimulate TRAIP ubiquitylation of Mcm7 (Fig. 5), despite TTF2 being present in S-phase nuclei.

It is likely that the remodeling of the replisome upon transition from S-phase to mitosis (Supplementary Table S1) additionally stimulates TRAIP activity. This may be through novel interactors in mitosis such as FancD2, Rad52, WRN, or ATR or their enzymatic activity. The mechanism may also be through eviction of some of the replisome components such as Mcm10, Donson, or Claspin, which may be blocking the ‘active’ docking of TRAIP within the S-phase replisome, or additional mitotic post-translational modifications of other replisome factors. Further studies will be required to decipher the full mechanism of TRAIP activation in mitosis.

Our findings that phosphorylation of TRAIP affects its ubiquitin ligase activity in *in vitro* self-ubiquitylation reactions (Fig. 3A) suggest that phosphorylation either drives conformational changes within TRAIP stimulating the E3 activity or stimulates interactions of TRAIP with other components required for the reaction: ubiquitin or E2. One possibility is that phosphorylation of TRAIP reorganizes its C-terminal disordered region (Supplementary Fig. S3F) allowing for much greater flexibility for TRAIP protein. The model of dimeric TRAIP structure looks very much like a crane with the double RING domains at the end of a flexible arm. The greater flexibility in the movement of the arm could allow TRAIP RING domains to reach more of its own lysines or more lysines on substrates around it within the host replisome (Fig. 5D).

We have shown here that the CDK phosphorylation of TRAIP is essential for TRAIP’s activity in mitosis in human cells (MiDAS, Fig. 4) and to support cell viability upon mild replication stress, but not in unperturbed cell cycle, as otherwise unchallenged cells can grow well with TRAIP-3A as the only form of TRAIP (Fig. 4B). This suggests that TRAIP-3A is a separation of function mutant of TRAIP, which can support S-phase functions of TRAIP (essential for cell viability [17]), but not the mitotic ones (MiDAS). It will be therefore a valuable tool in the future to detangle the impact of these two activities of TRAIP on cell viability and genome maintenance. As mutations of TRAIP lead to microcephalic dwarfism [19], deeper understanding of TRAIP activity and regulation will shed light on sources of development of this disorder.

## Supplementary Material

gkaf530_Supplemental_File

## Data Availability

The mass spectrometry proteomics data have been deposited to the ProteomeXchange Consortium via the PRIDE repository with the dataset identifier PXD058941.
