# Supplementary material for: CDK-driven phosphorylation of TRAIP is essential for mitotic replisome disassembly and MiDAS
Source: Nucleic Acids Res. 2025 Jul 10;53(13):gkaf530. doi: 10.1093/nar/gkaf530 (PMC12242765; doi:10.1093/nar/gkaf530)
Supplement: gkaf530_Supplemental_File [file gkaf530_supplemental_file.pdf]

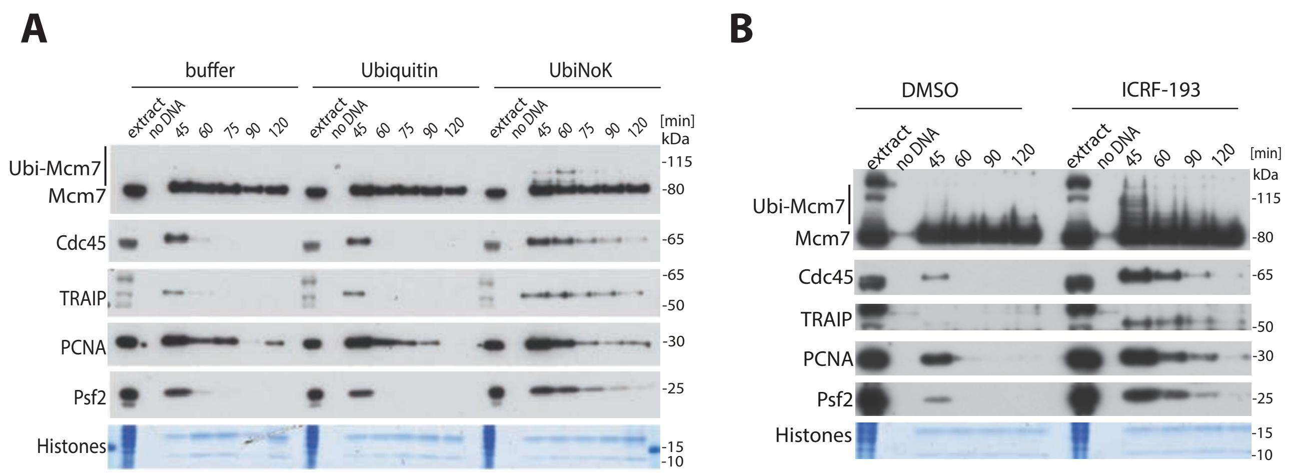

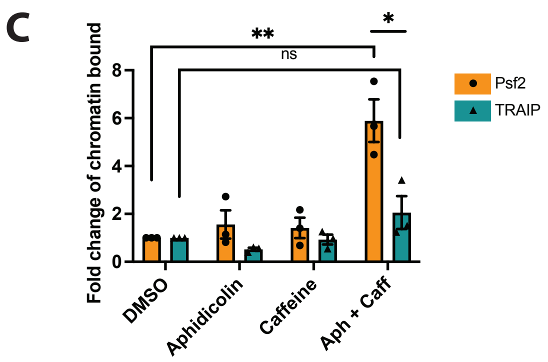

Supplementary Figure 1. (A) DNA replication was set up in *Xenopus* egg extract with optional addition of 6His-Ubi or 6His-UbiNoK and at the indicated times chromatin was isolated and chromatin bound factors resolved on SDS-PAGE and immuno-blotted with indicated antibodies. "No DNA" control served as chromatin specificity control, while Coomassie stained histones from the bottom of the gel serve as a loading and purity control. (n=3). **(B)** As in (A) but Topo2 inhibitor ICRF-193 was optionally added. (n=3). **(C)** Quantification of Psf2 and TRAIP chromatin binding from Figure 1B. n=3, mean with SEM presented.

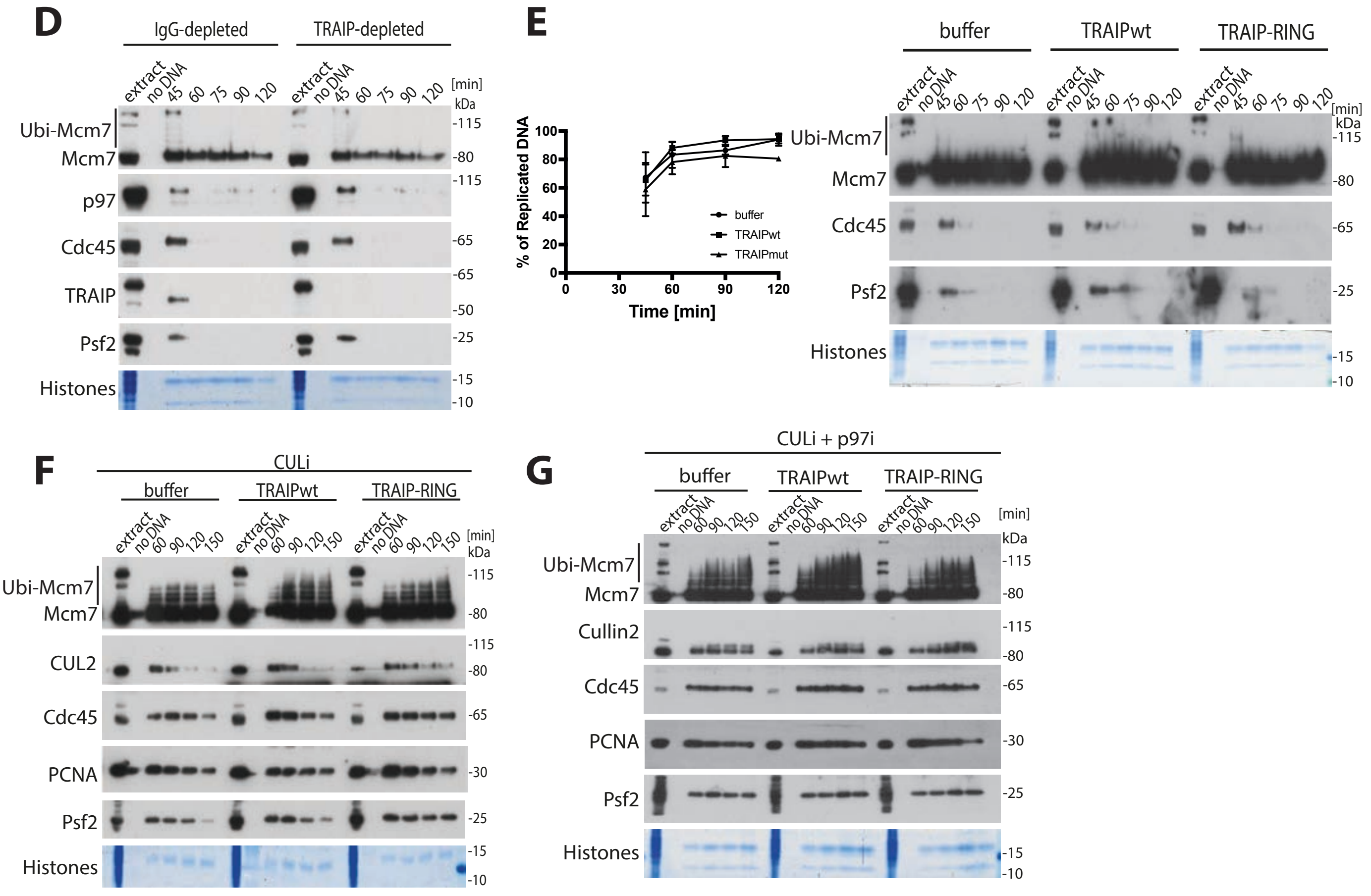

Supplementary Figure 1. (D) DNA replication was set up in IgG- or TRAIP-depleted egg extracts and at the indicated times chromatin was isolated and chromatin-bound factors resolved on SDS-PAGE and immunoblotted with indicated antibodies (n=3). **(E)** DNA replication was set up in egg extract with optional addition of TRAIPwt or TRAIP-RING mutant at a final concentration of 1.5 μM. Extract ability to synthesise nascent DNA was analysed by measuring incorporation of α-P32-dATP into DNA at indicated timepoints (n=3) (left). Chromatin fractions were isolated at indicated times and analysed by immunoblotting as before (n=3) (right). **(F, G)** DNA replication was set up as above, but with addition of CULi (MLN4924) (n=2) (F) and both CULi and p97i (NMS873) (n=2) (G). Chromatin fractions were isolated at indicated times and analysed by immunoblotting as before.

Supplementary Figure 1

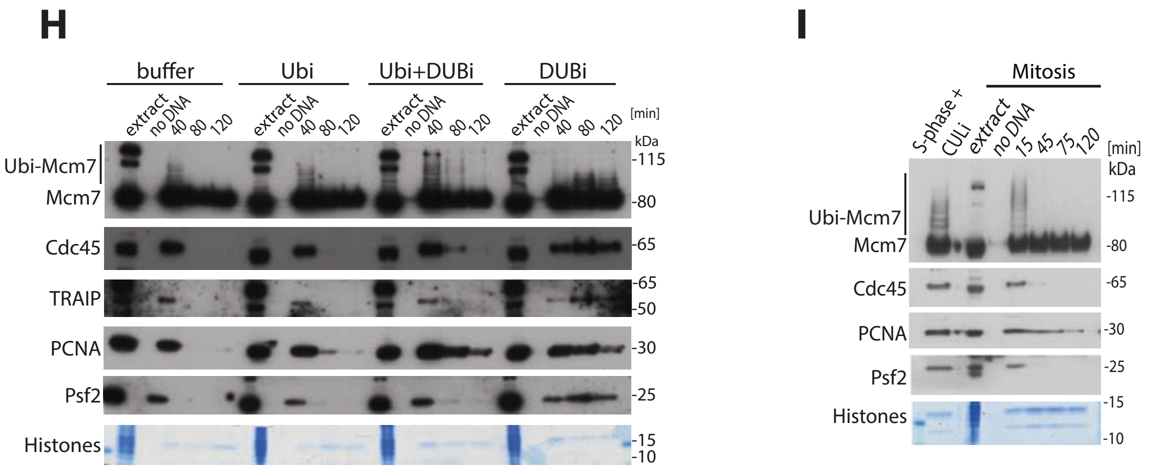

Supplementary Figure 1. (H) DUBi depletes free pool of ubiquitin. DNA replication was set up in egg extract with optional addition of ubiquitin and DUBi (Ubi-Vs) (n=2). Chromatin fractions were isolated at indicated times and analysed by immunoblotting as before. **(I)** DNA replication was completed in egg extract supplemented with CULi upon which CyclinA1DN was optionally added to the extract. Chromatin was isolated at S phase at 90 min and indicated times after CyclinA1DN addition. The Mcm7 ubiquitylation of Mcm7 and replisome unloading was analysed as earlier (n>3).

Supplementary Figure 2

A

```
CLUSTAL O(1.2.4) multiple sequence alignment

Human               ------MPIRALCTICSDFFDHSRDVAAIHCGHTFHLQCLIQWFETAPSRTCPQCRIQVG    54
M. musculus         ------MPIRALCTICSDFFDHSRDVAAIHCGHTFHLQCLIQWFETAPSRTCPQCRIQVG    54
X. laevis           ------MPIRAYCTICSDFFDNARDVAAITCGHTFHQECLLQWFHSAPHRTCPQCRIQVS    54
D. rerio            ------MPIRAYCTICSDFFDNSKDVAAIHCGHTFHYSCLLQWFQSAPNKTCPQCRKQVS    54
C. elegans          MTSQPTSSLQGSCSICFEDLKQNDKISAIVCGHIYHHGCISQWIAT--KRQCPSCRRTVP    58
D. melanogaster     -------MLNLNCVICAELFGQADEVFATVCGHMFHHNCLNQWLDR--SKTCPQCRNKCT    51
                            :.  * ** : : :  .: *  *** :*  *: **:     : **.**

Human               KRTIINKLFFDLAQEEE-NVLDAEFLKNELDNVRAQLSQKDKEKRDSQVIIDTLRDTLEE    113
M. musculus         KKTIINKLFFDLAQEEE-NVLDAEFLKNELDSVKAQLSQKDREKRDSQAIIDTLRDTLEE    113
X. laevis           SRQIINKLFFDIGGEEE-TVLDAESLKNEVDRIKASLLVKEKEKRECQGLVDSLREMLDV    113
D. rerio            TRHIINKLFFDIAPEDDGAPVDPESLQNELDRMKAVLSEKEKEWREKQKTVDTLKDTIEK    114
C. elegans          KNGFVEKLFFDVQRMGGEAEKPPE-IDYREEHYKLSTSLKV-----EQEKLGTLNTENKN    112
D. melanogaster     TRNIF-RVYFNLANLDV-SHIDVGSLQEQLDNAMLSMKMVEKERNKDEQQIRDLKETQKK    109
                    .. :. :::*::             :. . :                :  :  *.   .

Human               RNATVVSLQQALGK----AEMLCSTLKKQMKYLEQQQDETKQAQEEARRLRSKMKTMEQI    169
M. musculus         RNATVESLQNALNK----AEMLCSTLKKQMKFLEQRQDETKQAREEAHRLKCKMKTMEQI    169
X. laevis           RNVTIQSQQKELGD----MEMLCSTLKKQIKFLDKQQSETKAAKDEARKLRNKLKTMESI    169
D. rerio            QKKDLDKVRKEIGD----KEMLCSVLRKQMNFLESQKNEGQAAKEEAKRLRVKMKTYESL    170
C. elegans          LKDTVKSLEKKIIREKDKYRQEIPKLQATINHLTISSEETAYLKRELQESKNRLKTCEFY    172
D. melanogaster     CLKTIAGLEQKVQK----KDFLISSYVEQIGVLKSDAHVVDGLRKENKTLKSQIQSMEGI    165
                        :   .: :                 :  *          : * :  : :::: *

Human               ELLLQSQRPEVEEMIRDMGVGQSAVEQLAVYCVSLKKEYENLKEARKA----SGEVADKL    225
M. musculus         ELLLQSQRSEVEEMIRDMGVGQSAVEQLAVYCVSLKKEYENLKEARKA----TGELADRL    225
X. laevis           EVLLQSQRSEVEEMIRDMGSGQAAVEQLAIYCVSLKKEYENLKEVRKS----SAEMTEKL    225
D. rerio            DVVLQGQRSEVEAMITDMGVGQSAVEQLSIYCISLKKEYDNLKGSLKS----SNEMCEKL    226
C. elegans          KIL-TVHSSEADKQLGEYLKKNGNLDT--------EKFFQLMKSTNK------------    210
D. melanogaster     SAILAAGSADADRLLKNEAD----PHVLANWVSTLKRELRQCESKKTELRNVVKVVQNDL    221
                    . :      :.:  : :        .        ::     :   .

Human               RKDLFSSRSKLQTVYSELDQAKLELKSAQKDLQSADKEIMSLKKKLTMLQE---------    276
M. musculus         KKDLVSSRSKLKTLNTELDQAKLELRSAQKDLQSADQEITSLRKKLMILQG---------    276
X. laevis           RKELFSSNHKAQKAELELTKVREELSASQKELHSADKEIMSLKKKVEFLQK---------    276
D. rerio            KREMISSNSKLQKATSETNRTKEDMKALQKDLSSADKEITSLKKKVEILQR---------    277
C. elegans          --DLTDKR---REAAKEIEQLKMEVQSLKRAAQEDAAIKKTLKKTVLDLRE---------    256
D. melanogaster     RKEIELKR-KL----------EERVSHLESDLYQAQEKLQAFENKTAYLDSPNASCGLNS    270
                      ::  ..             .  :   :    .     ::.:.   *

Human               ----------TLNLPPVASETVDRLVLESPAPVEV-NLKLRRPSFRDDIDLNATFDVDTP    325
M. musculus         ----------TLSLPPATNETVSRLVFESPAPVEMMNPRLHQPPFGDEIDLNTTFDVNTP    326
X. laevis           ----------TLTTPTASNEAISRLIFESPAPIGLERPKLRPPMMGNDINLDVTFDIDTP    326
D. rerio            ----------TLSTPTRTNEAISRLVFESPAPLELKPPGHGHMTDSQEIDLNLTFDINTP    327
C. elegans          --------RANVDTPINNKRLRDVLETETPPPAKRKSMGFDESSQMIDPDGELSFFKQN-    307
D. melanogaster     NILALKREERRTTISPTVKENIKRIEESTSPYLNIKSSSV-----GLAHLLNTKGN----    321
                                      ..  . :  .:                      : .

Human               PARPSSSQHGYYEKLCLE-KSHSPIQDVPKKICKG-----PRKESQLSLGG---------    370
M. musculus         PTQTSGSQHCLPKKLCLE-RARSPMQNVLKKVHKV-----SKPESQLSLGG---------    371
X. laevis           EHNTQKSVVAPFKKMKFD-NKEHPLSSPTKNPLQE-----SK--GLMSWAG---------    369
D. rerio            EHVEKKPVQVPKK-MRLD-PSVS---SSTQ--------------NTENTQG---------    359
C. elegans          -------------------ENRTPVTSVPSTSKAVLPFNFDDDEDDEYFKTPKIAEKKKK    348
D. melanogaster     --------------IGLAKSKISPIKGVGG---------------GVSMTS---------    343
                                              .

Human               QSCAGEPDEELVGAFPIFVRNAILGQKQPKRPRSESSCSKDVVRTGFDGLGGRTKFI---    427
M. musculus         QRCVGELDEELAGAFPLFIRNAVLGQKQPNRTTAESRCSTDVVRIGFDGLGGRTKFI---    428
X. laevis           GRTGA--DEDDDLTLPSFIKNSLLHKK-PVGSLLGLRQNTGAVRTGFDGLGGRTKFI---    423
D. rerio            RNNGR--VKDDDVMMGSLFRNSLLFRK-NAFGSMLDPCKSGTVRSGYDGLGGRAKFI---    413
C. elegans          LPEAMAPVSEDSFDFDIAVPQSIINRI-PAKTTAQP----AKKYPKIPNLSAKTSSKPTE    403
D. melanogaster     G-TIRKTSSDLSEKYSIFKKPRLLLGSSS-SSAL---TATTGSNFVYNGMGGSEKVDPFA    398
                            .:            ::                        .:..  .

Human               QPTD---TVMIRPLPVKPKTKVKQRVRVKTVPSLFQAKLDTFLWS---------------    469
M. musculus         QPRD---TTIIRPVPVKSKAKSKQKVRIKTVSSASQPKLDTFLCQ---------------    470
X. laevis           QPSN---LTEIRPLHQKMKRKKVSRPTACTSSLANQPRLEDFLK----------------    464
D. rerio            QPSP---LSEIRPLVLKSKRKKVSRPVTSKPTS-SLTTLDGFLE----------------    453
C. elegans          APKTEN-VLKIKSKS----QEIAQKPQKS-------TRISSFFSRTTSSTSNLTEYVILD    451
D. melanogaster     QRAEEEGLSTIRSSTAL-SRNVNQRLKAG--------SLRNFNLGK--------------    435
                              *:        :  .:             :  *
```

* Identical residues (perfect match)
: Conserved substitutions (similar properties)
. Weakly similar substitutions

B

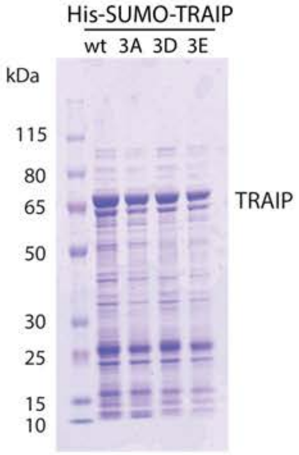

C

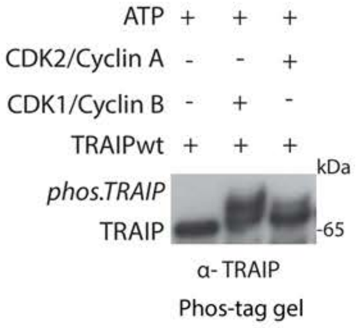

Supplementary Figure 2. (A) Sequence aligment of TRAIP from different species. The CDK concensus / phosphorylated residues are marked **(B) Purified His6-SUMO-TRAIPwt and mutants.** *X.laevis* His6-SUMO-TRAIPwt and indicated mutants were expressed in *E.coli* and affinity purifed as described in methods. Samples of purified proteins were separated on SDS-PAGE and stained with Coomassie blue (n>3). **(C) Mitotic CDKs can phosphorylate His6-SUMO-TRAIPwt.** *In vitro* phosphorylation reactions were set up with indicated CDK/cyclins combinations, reactions resolved on Phos-tag gel and immunoblotted with α-TRAIP antibodies (n=2).

D

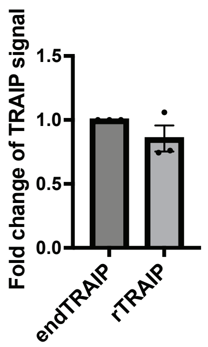

E

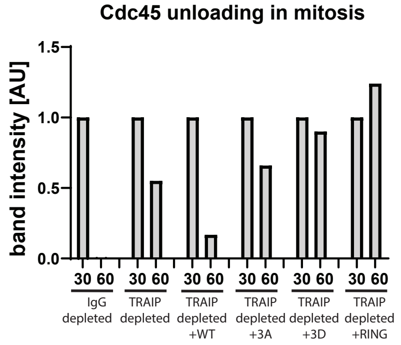

F

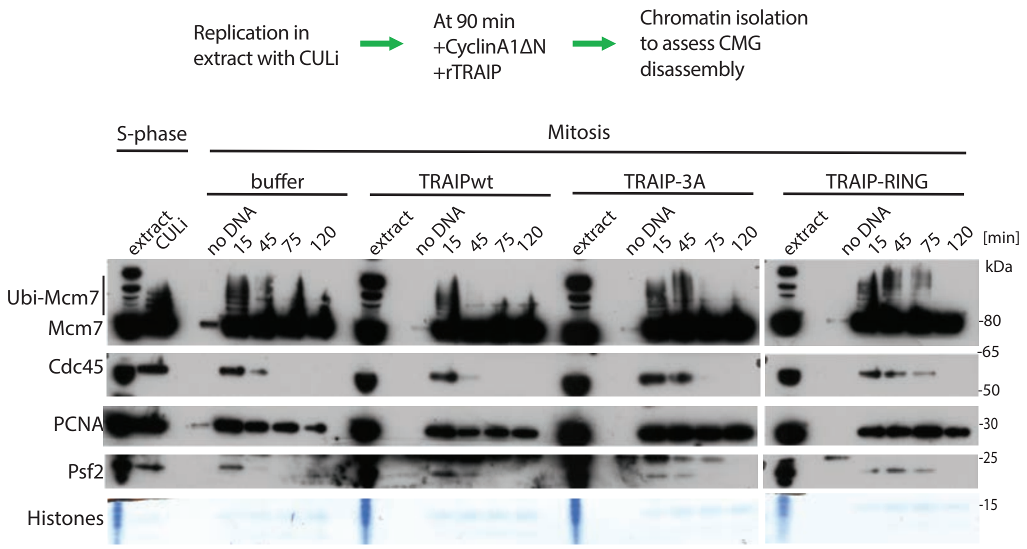

Supplementary Figure 2. (D) The level of recombinant TRAIP added to TRAIP-depleted extract closely resembles the level of endogenus TRAIP. Western blot signal of endogenous TRAIP and rTRAIP added to the TRAIP-depleted extract was quantified in 3 experiments. The signal was normalised to endogenous TRAIP and mean plotted with SEM. **(E) Phosphorylation of TRAIP is essential for mitotic replisome disassembly.** Quantification of Cdc45 signal from an experiment analogous to one in Figure 4A but performed with independent TRAIP-depleted extract. **(F) Addition of high concentration of TRAIP-3A delays mitotic replisome unloading.** Replication reaction was set up in egg extract in presence of CULi. After completion of DNA synthesis (90 min) CyclinA1DN was added to the extracts to drive them into mitosis. At the same time recombinant TRAIPwt or indicated mutants were added. Chromatin was isolated at indicated times after CyclinA1DN addition, to assess CMG disassembly.

A

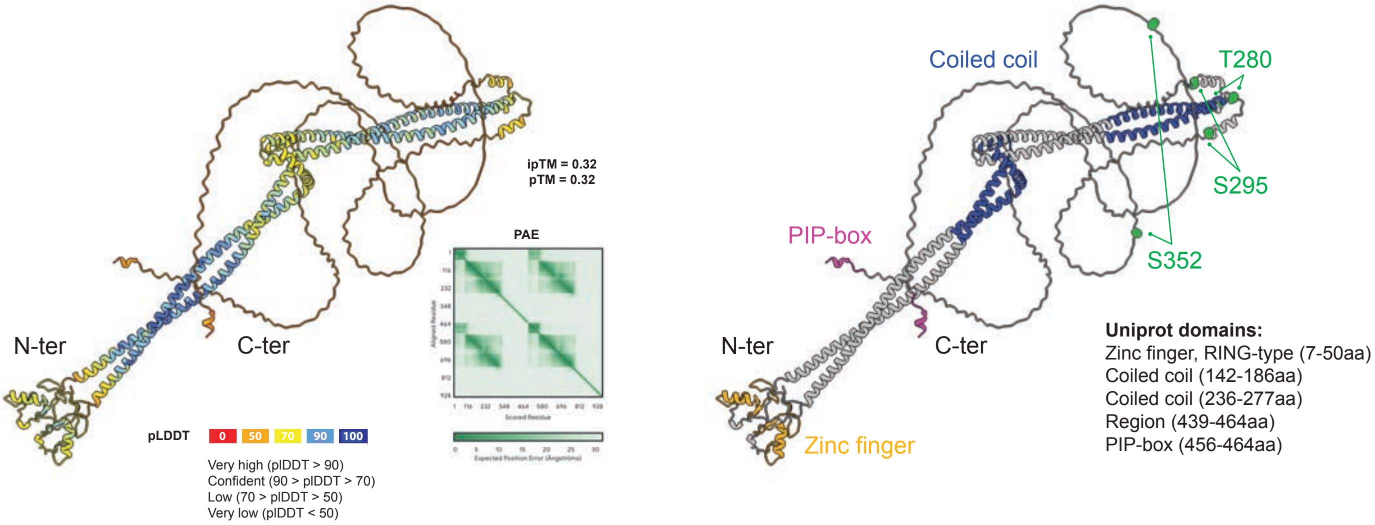

B

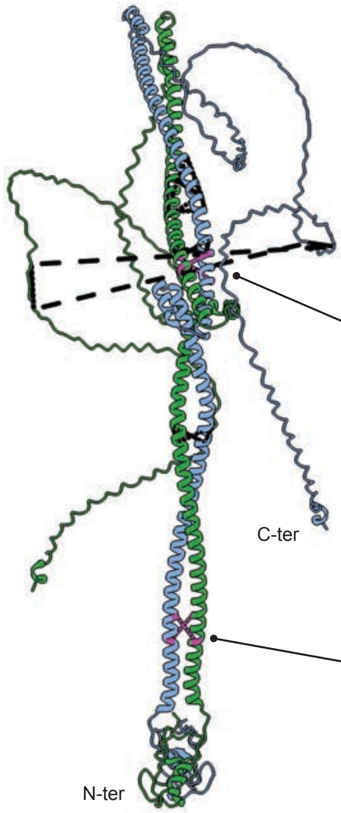

	Peptides	Pos.1	Pos.2
1)	ASLLVKEK-IKASLLVK-a6-b2	93	87
2)	ELGDMEMLCSTLKK-QIKFLDK-a13-b3	136	140
3)	DXGSGQAAVEQLAIYCVSLKK-EYENLKEVR-a20-b6	205	212
4)	EYENLKEVR-KEYENLK-a6-b1	212	206
5)	EYENLKEVR-KSSAEMTEK-a6-b1	212	216
6)	SSAEMTEKLR-KELFSSNHK-a8-b1	224	227
7)	LRKELFSSNHK-AQKAELELTK-a3-b3	227	238
8)	AQKAELELTK-KELFSSNHK-a3-b1	238	227
9)	TGADEDDDLTLPSFIKNSLLHK-KPVGSLLGLR-a16-b1	387	394

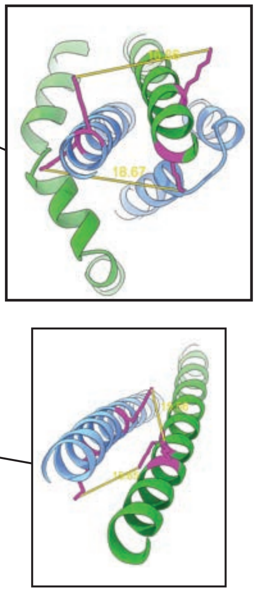

Supplementary Figure 3. (A) AlphaFold modellingof dimer TRAIP. AlphaFold model of dimer TRAIP with best prediction score in blue and worse prediction score in orange (left). Dimer structure of TRAIP with TRAIP domains (as per Uniprot) indicated. The 3 identified phosphorylated sites are indicated. **(B) TRAIP forms a dimer.** Crosslinking mass spectrometry analysis of recombinant His6-SUMO-TRAIP. The table lists the identified crosslinked peptide pairs with unambiguous inter-molecular links highlighted in purple. X designates an oxidized methionine residue. The cross-linked lysine positions (#) in the first and second peptide are designated with a# and b# after the peptide sequences. Numbering of the amino acid positions is according to the wild-type sequence.

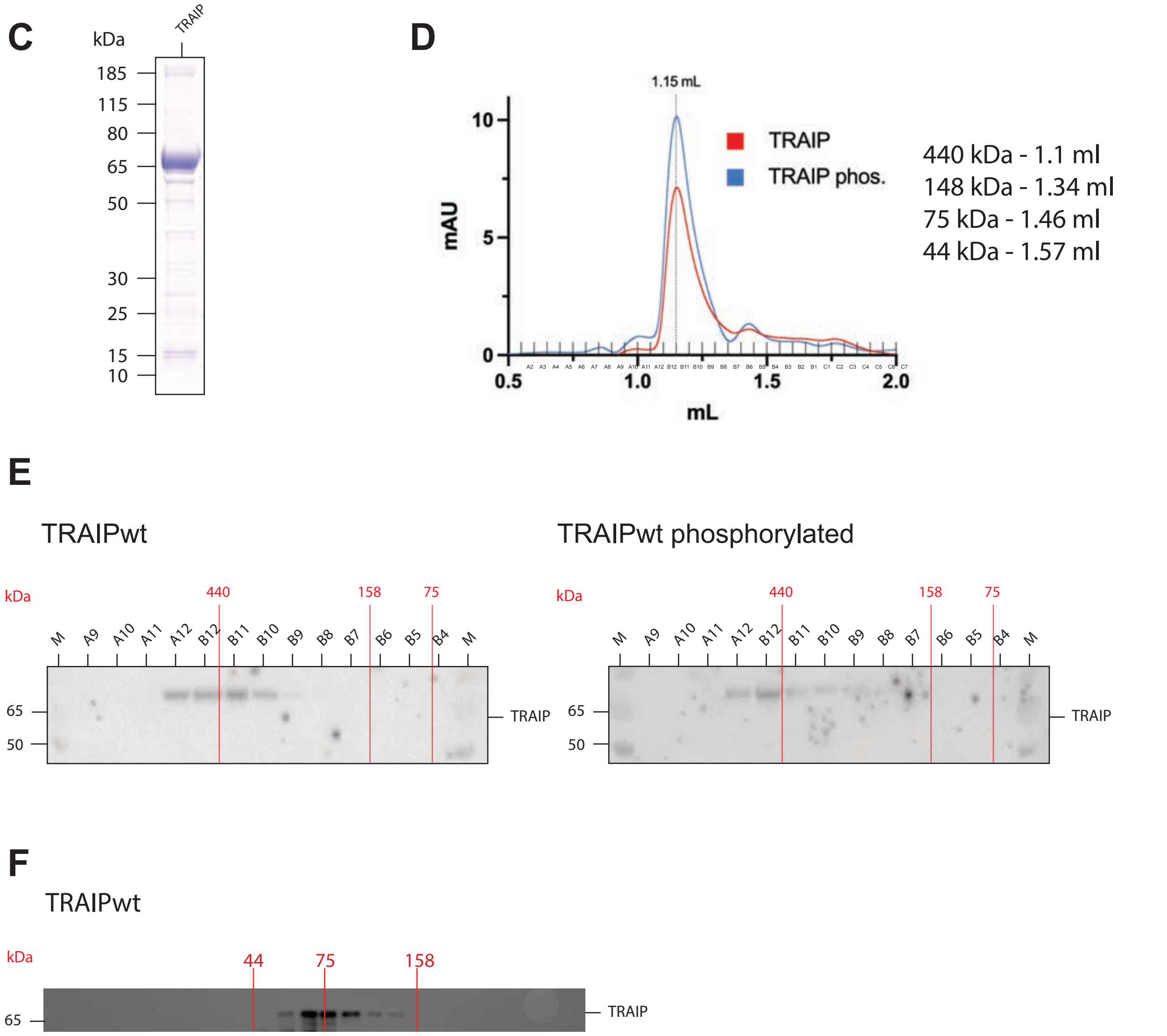

Supplementary Figure 3. (C) Recombinant *X.laevis* His6-SUMO-TRAIP used in analyses resolved on SDS-PAGE and stained with coomassie. **(D) Dimer TRAIP has elongated structure.** His6-SUMO-TRAIP either non-phosphorylated or phosphorylated *in vitro* with CDK1/cyclin B was resolved on Superdex 200 gelfiltration column. UV absorbance peaks corresponding to TRAIP and phosphorylated TRAIP. **(E)** Aliquotes of each indicated gel filtration fraction from (B) were resolved on SDS-PAGE and immuno-blotted with α-TRAIP antibodies. **(F)** His6-SUMO-TRAIP was centrifuged through a glycerol gradient calibrated with indicated markers. The gradient was seprated into 20 fractions and aliquotes of each resolved on SDS-PAGE and immunoblotted with α-TRAIP antibodies.

G

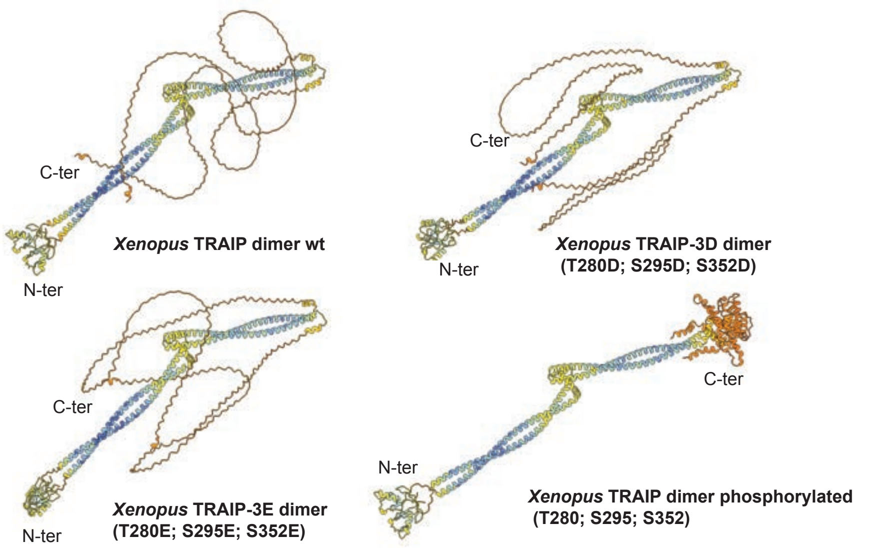

H

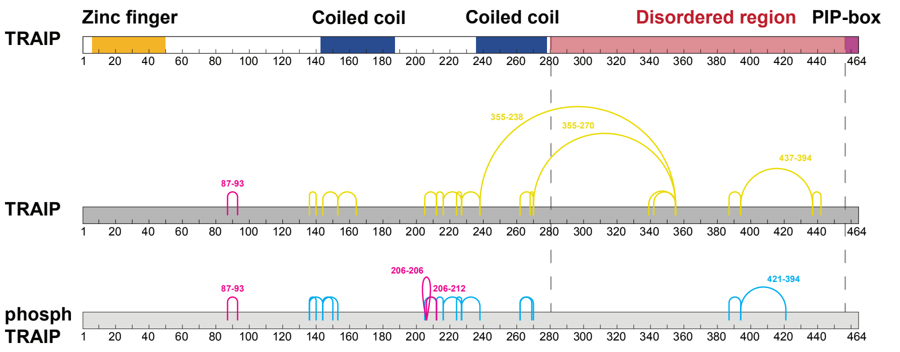

Crosslinks in same protein or potentially between two different molecules of the same protein (Homomultimeric).

Crosslinks with overlapping peptides. Cannot be the same molecule, so it is either between two different molecules of the same protein or a mis-identification.

I

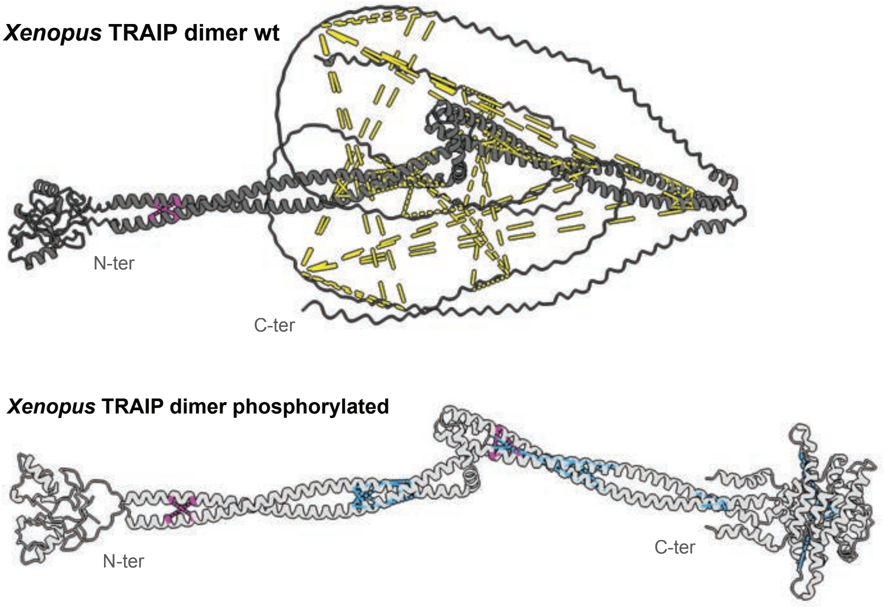

	Peptides	Pos.1	Pos.2
1	ASLLVKEK—IKASLLVK	93	87
2	ELGDMEMLCSTLKK—QIKFLDK	136	140
3	FLDKQQSETK—AAKDEAR	144	153
4	LKTMESIEVLLQSQR—AAKDEAR	164	153
5	DMGSGQAAVEQLAIYCVSLKK—EYENLKEVR	205	212
6	EYENLKEVR—KSSAEMTEK	212	216
7	SSAEMTEKLR—KELFSSNHK	224	227
8	LRKELFSSNHK—KSSAEMTEK	227	216
9	AQKAELELTK—KELFSSNHK	238	227
10	ELHSADKEIMSLKK—KVEFLQK	262	270
11	EIMSLKK—KVEFLQK	268	270
12	EHPLSSPTKNPLQESK—MKFDNK	355	342
13	EHPLSSPTKNPLQESK—SVVAPFKK	355	339
14	EHPLSSPTKNPLQESK—AQKAELELTK	355	238
15	EHPLSSPTKNPLQESK—KVEFLQK	355	270
16	TGADEDDDLTLPSFIKNSLLHK—KPVGSLLGLR	387	394
17	FIQPSNLTEIRPLHQKMK—KPVGSLLGLR	437	394
18	FIQPSNLTEIRPLHQKMK—KVSRPTACTSSLANQPR	437	442

	Peptides	Pos.1	Pos.2
1	ASLLVKEK—IKASLLVK	93	87
2	ELGDMEMLCSTLKK—QIKFLDK	136	140
3	ELGDMEMLCSTLKK—FLDKQQSETK	136	144
4	FLDKQQSETK—AAKDEAR	144	153
5	FLDKQQSETK—QQSETKAAK	144	150
6	DMGSGQAAVEQLAIYCVSLKK—EYENLKEVR	205	212
7	KEYENLK—KEYENLK	206	206
8	EYENLKEVR—KSSAEMTEK	212	216
9	EYENLKEVR—KEYENLK	212	206
10	SSAEMTEKLR—KELFSSNHK	224	227
11	KELFSSNHK—KSSAEMTEK	227	216
12	AQKAELELTK—KELFSSNHK	238	227
13	ELHSADKEIMSLKK—KVEFLQK	262	270
14	ELHSADKEIMSLK—KKVEFLQK	262	269
15	TGADEDDDLTLPSFIKNSLLHK—KPVGSLLGLR	387	394
16	TKFIQPSNLTEIRPLHQK—KPVGSLLGLR	421	394

Supplementary Figure 3. (G) AlphaFold 3 modelling of phosphorylated TRAIP. AlphaFold 3 model of dimeric *X.laevis* TRAIP with the 3 identified phosphorylated sites either in unmodified form, changed to D or E or phosphorylated *in silico*. High confidence of prediction in blue, low confidence of prediction in orange. **(H) TRAIP and phosphorylated TRAIP diagrams.** Cross-links for TRAIP (yellow) and phosphorylated TRAIP (cyan) are mapped on the protein diagrams. Cross-links with overlapping peptides indicating that TRAIP can homodimerize are in magenta. Diagrams obtained with xiVIEW. **(I) TRAIP and phosphorylated TRAIP cross-links.** The detected cross-links mapped on the AlphaFold 3 models of TRAIP dimer wt (dark grey) and TRAIP dimer phosphorylated (T280; S295; S352) (light grey), alongside with the list of cross-links. Long distance cross-links are underlined. Figure created with ChimeraX and XMAS.

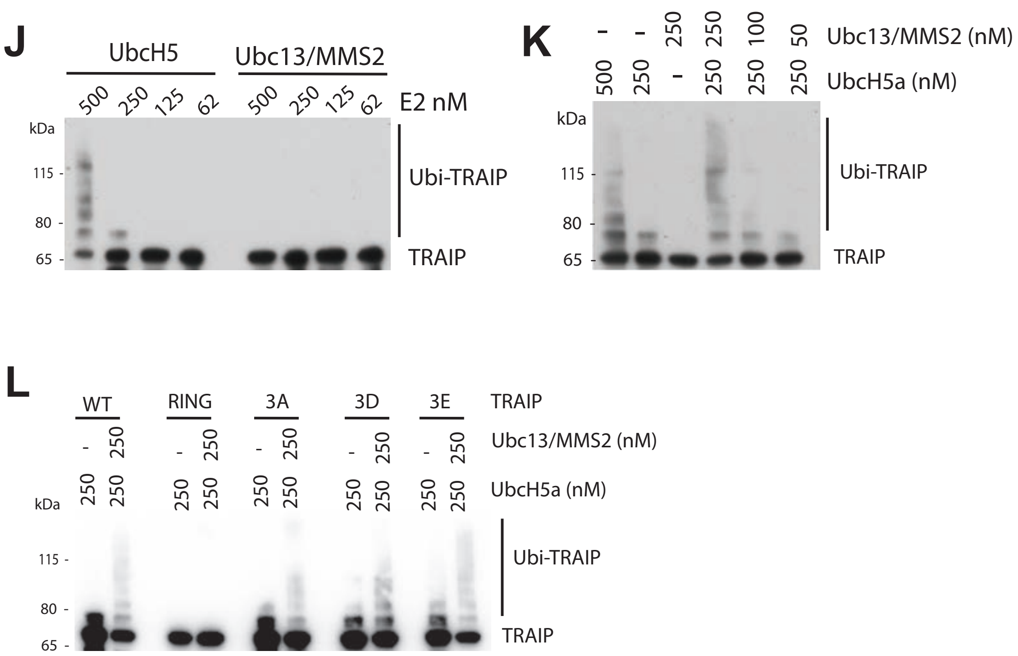

Supplementary Figure 3. (J) TRAIP can autoubiquitylate in presence of UbcH5. His6-SUMO-TRAIP autoubiquitylation reaction was set up in presence of E1, ubiquitin, ATP and indicated concnatrations of E2 enzymes UbcH5 or Ubc13/MMS2. The ubiquitylation reactions were run on SDS-PAGE gels and immunoblotted with α-TRAIP antibodies. **(K) Combination of UbcH5 and Ubc13/MMS2 is most efficient at autoubiquitylation of TRAIP.** As in (A) but indicated combinations of E2s used. **(L) All mutants of TRAIP (apart from RING mutant) are able to autoubiquitylate.** Indicated mutants of His6-SUMO-TRAIP were analysed for autoubiquitylation activity as in (B).

Supplementary Figure 4

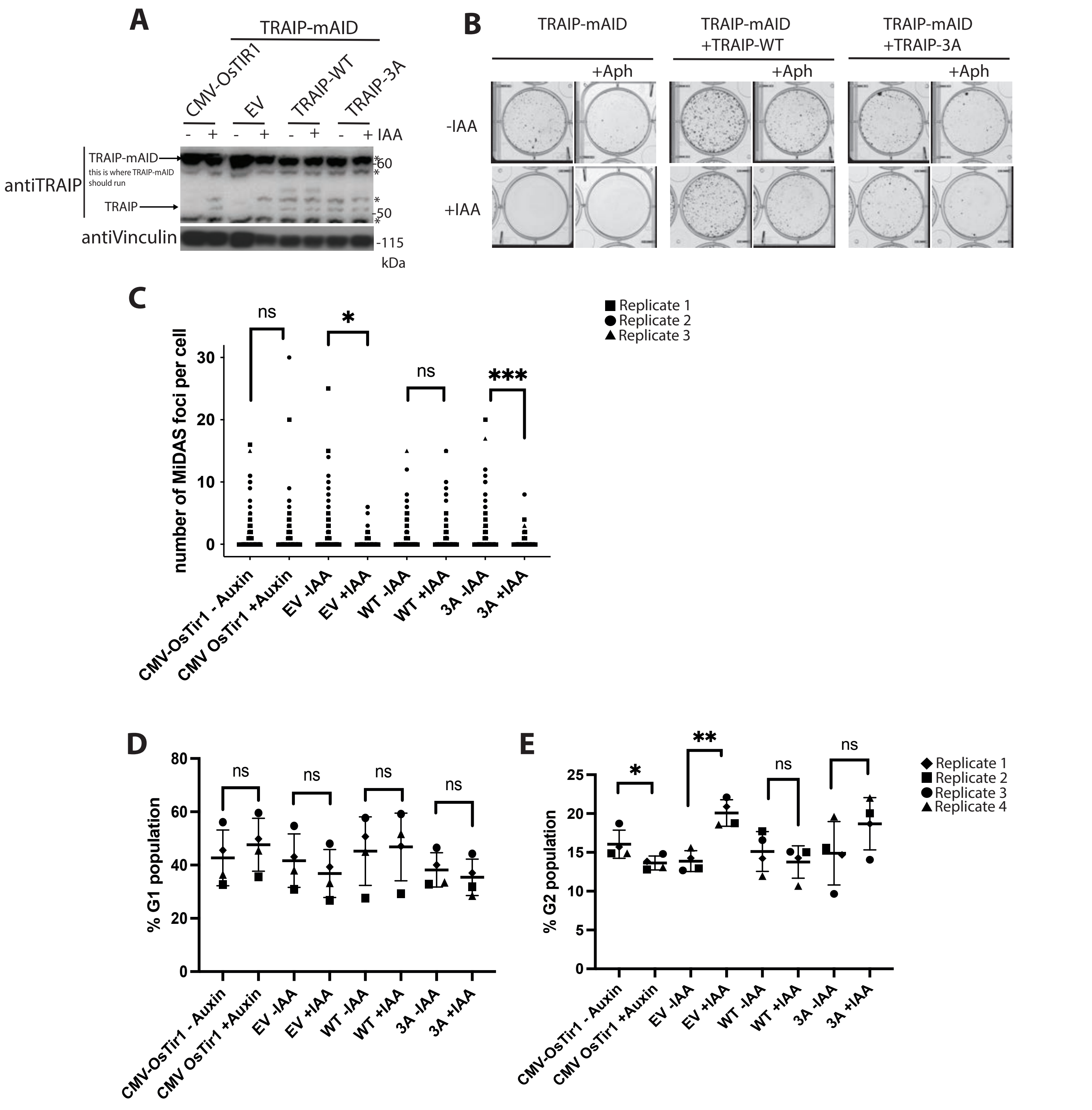

Supplementary Figure 4. (A) The expression level of TRAIP-mAID and endogenous TRAIP, TRAIPwt and TRAIP-3A were assessed by immunoblotting in whole cell extract (WCE), using a-TRAIP antibody, arrows point at where TRAIP endogenous, TRAIP-EV/WT/3A (referred as TRAIP in figure) run and TRAIP-mAID should run, *indicate aspecific bands (n=3). **(B)** Examples of the colony formation assay quantified in Figure 4B**. (C)** number of MiDAS foci per cell in samples optionally treated with auxin , non parametric student's t-test (n=4). **(D)** Percentage of G1 popylation and **(E)** percentage of G2/M population in asynchronous cells optionally treated with auxin, non parametric student's t-test (n=4).

Supplementary Figure 5

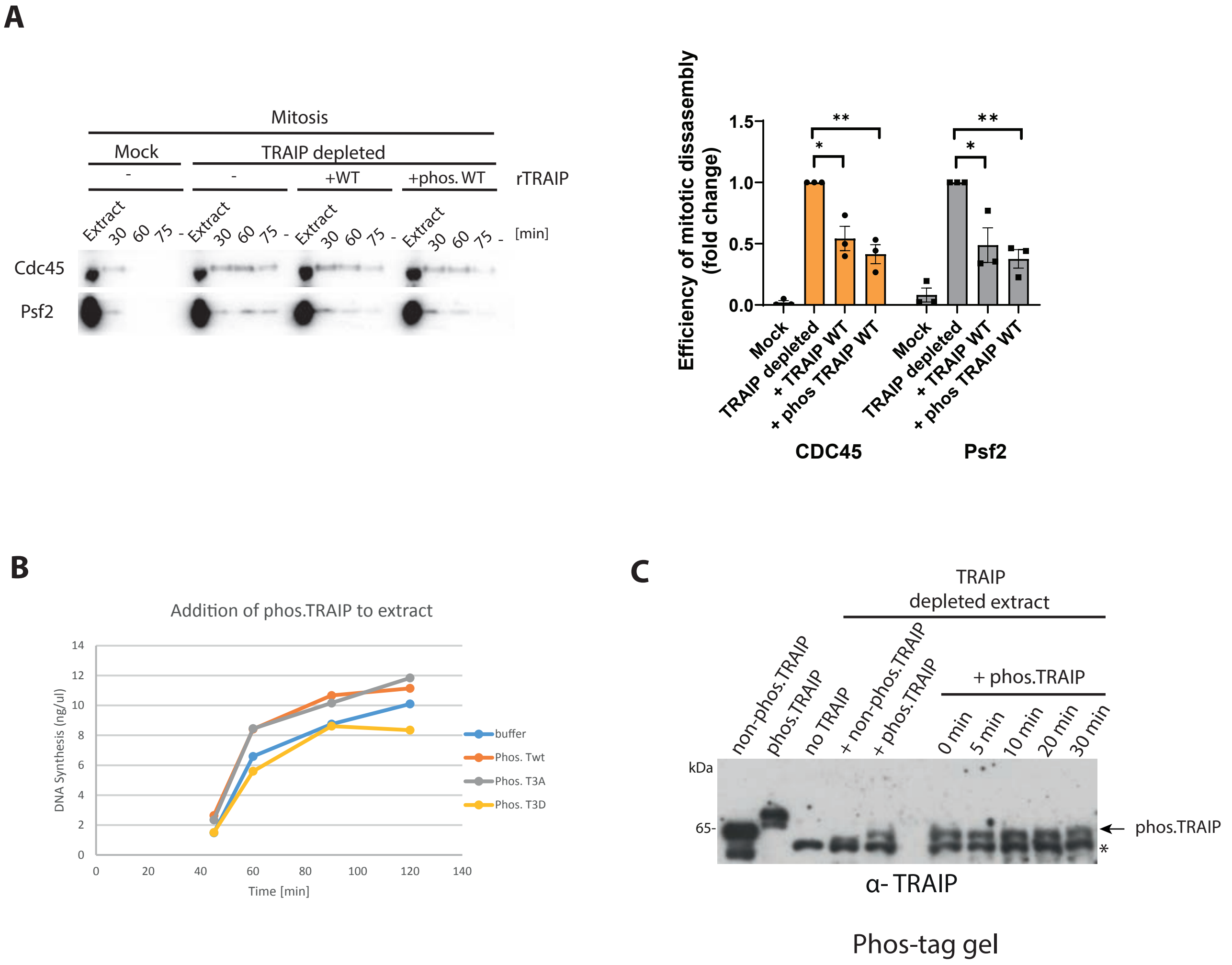

Supplementary Figure 5. (A) Phosphorylated TRAIP added to TRAIP-depleted extract dissasembles CMGs from chromatin. TRAIP-depleted extract was suplemented with TRAIP WT or phosphorylated TRAIP WT and chromatin isolation in mitosis was performed. CMGs dissasembly was assessed by measuring the intensity of Cdc45 and Psf2 bands in Western Blot. The results were visualised as a ratio of band intensity at 75/30 min, normalised against the corresponding ratio in the TRAIP-depleted extract only, (n=3, mean with SEM, *$p<0.05$, **$p<0.005$). **(B) Addition of phosphorylated TRAIP in S-phase does not affect extract's ability to synthesise nascent DNA.** DNA replication reaction was set up in egg extract optionally supplemented with phosphorylation buffer, phosphorylated His6-SUMO-TRAIP WT or indicated mutants. The incorporation of α-32P-dATP into nascent DNA was measured at indicated times (n=1). **(C) Phosphorylated TRAIP remains in phosphorylated form once added to S-phase extract.** TRAIP-depleted extract was supplemented with phosphorylated His6-SUMO-TRAIP wt. Samples taken during replication reaction at indicated times were resolved on Phos-tag gel and visualised by immunoblotting with α-TRAIP antibody (n=2).
* indicates non-specific band.

Supplementary Table 1. Replisome interacting proteins change abundance in S-phase vs mitosis. Proteins identified in Mcm3 IP in S-phase (replisome retained on chromatin due to CULi and p97i treatment) and in mitosis (replisome retained on chromatin due to Culi and p97i treatment and mitosis induced through CylinA1ΔN addition). Published previously in Sonneville et al 2017 and Priego Moreno et al 2019. No of peptides identified (normalised to total number of Mcm2-7 complex) and fold difference Mitosis/S-phase is presented. Only proteins with at least 50 peptides identified in either dataset are presented.

Protein name	S-phase Mcm3 IP [no of pept]	Mitosis Mcm3 IP [no of pept]	fold change
H1	1	123	123
FancD2	1	100	100
WRN	1	92	92
KIF1	1	53	53
NCAPD2	1	52	52
LOC398587	7	135	19.3
ATR	8	136	17
Rad52	32	250	7.8
YBX2-A	10	54	5.4
VASP	25	53	2.12
Ubi	128	247	1.9
SMC1	31	53	1.7
Mcm3	1225	1388	1.1
Mcm5	1005	1132	1.1
DNMT1	57	60	1.1
Mcm4	1052	1079	1
Top2a	535	533	1
Mcm7	986	922	0.9
Mcm2	1072	984	0.9
H3.2	96	83	0.9
GINS3	61	52	0.9
Mcm6	950	768	0.8
SSRP	256	205	0.8
Top2b	77	61	0.8
GINS1	69	53	0.8
UBTF-1	96	62	0.6
Spt16	504	322	0.6
H4	81	51	0.6
Timeless	190	106	0.6
Cdc45	308	169	0.5
Baz1b	152	74	0.5
actin	308	134	0.4
EEF2	104	35	0.3
Pol eB	115	34	0.3

Pol e 1	605	154	0.3
And1	312	78	0.2
LRR1	51	11	0.2
CABIN1	92	16	0.2
KPNA7	95	14	0.1
SMARCA5	367	54	0.1
Cul2	138	4	0.02
HIRA	116	2	0.02
TCF20	62	1	0.02
UbtfB	67	1	0.01
PHF14	71	1	0.01
Donson	76	1	0.01
PARP1	79	1	0.01
Claspin	80	1	0.01
ORC1	105	1	0.01
Ctf18	192	1	0.01
Mcm10	287	1	0.003
